# Sounds as taxonomic indicators in Holocentrid fishes

**DOI:** 10.1038/s44185-024-00064-4

**Published:** 2024-11-05

**Authors:** Marine Banse, Estelle Bertimes, David Lecchini, Terry J. Donaldson, Frédéric Bertucci, Eric Parmentier

**Affiliations:** 1 Laboratoire de Morphologie Fonctionnelle et Evolutive, Université de Liège, 4000 Liège, Belgium; 2 PSL University, EPHE-UPVD-CNRS, USR 3278 CRIOBE, Moorea, French Polynesia; 3 Laboratoire d’Excellence “CORAIL”, 58 avenue Paul Alduy, 66860 Perpignan, France; 4 University of Guam Marine Laboratory/Guam EPSCoR, UOG Station, Mangilao, Guam 96921 USA; 5 Unité Mixte de Recherche MARine Biodiversity, Exploitation and Conservation, University of Montpellier, Centre National de Recherche Scientifique, Institut Français de Recherche pour l’Exploitation de la Mer, Institut de Recherche pour le Développement, Sète, France

**Keywords:** Biodiversity, Evolution, Zoology

## Abstract

The species-specific character of sounds in the animal kingdom has been extensively documented, yet research on fishes has predominantly focused on a limited number of species, overlooking the potential of acoustic signals to reflect broader taxonomic ranks. In this study, we analyzed acoustic data of hand-held sounds from 388 specimens spanning 5 genera and 33 species within the family Holocentridae, with the objective of evaluating the use of sound characteristics for taxonomic discrimination across various levels (subfamily, genus, species). Sounds could be indicative of grouping. Taxa discriminability depends on taxonomic level; the higher the taxonomic level, the better the discrimination of taxa based on sounds. Analogous to the role of morphological traits in taxonomic delineation, this research corroborates the utility of acoustic features in identifying fish taxa across multiple hierarchical levels. Remarkably, certain holocentrid species have evolved complex sound patterns characterized by unique temporal arrangements where pulses are not continuous but emitted in blocks, facilitating the exploitation of the acoustic space.

## Introduction

The use and importance of acoustic signals to communicate in many animal groups have been known for several decades. Studies of mammals, including bats^1,2^, primates^3,4^ and cetaceans^5^, birds^6–8^, frogs^9^, and orthopteran insects^10^ have reported that calls could be used in species discrimination. Similarly to those well-known taxa, sound production can also play a critical role in different social interaction contexts in teleost fishes^11^. These include territory defense^12^, warning calls, and predator signaling^13^, including mobbing^12,14^, aggressive interactions^15–17^, acoustically-mediated cleaning symbiosis^18^, and reproduction with courtship, gamete release and spawning^19,20^. During courtship, the use of specific calls could act as a prezygotic barrier that prevents confusion among species^21^. Moreover, sounds might communicate useful information on male quality or condition to a receiver^22^. In fishes, sound dominant frequency, amplitude, fatigue resistance, pulse period and calling activity can be informative of fitness or body size^22^.

In teleosts, the species-specific character of sounds has been investigated in several taxa^23–31^. Although species recognition information is not always encoded in the sounds, as observed in the loricariid catfish genus *Hypostomus*^29^, most of these studies reported that species could be identified based on their acoustical features. Moreover, hybrids produce sounds whose characteristics are midway between those of parent species which reinforces the species-specific and innate signature hypothesis on acoustic signals^32^. However, although statistical differences were found between the calls produced by closely related species, these studies are restricted to the comparison of a few species within a taxon, and playback experiments have almost never been conducted to test whether fish are capable of such discrimination. To the best of our knowledge, this type of experiment has been conducted only on the pomacentrid genus *Stegastes*^33,34^. While species may show a preference for sounds produced by individuals of the same species, the sounds themselves do not seem to act as a barrier.

The ability to produce sounds has evolved approximately 33 times during the history of actinopterygian fishes^35^. As a result, fish taxa produce sounds using several sound-producing mechanisms^11,36^. If phylogenetically close species share a common sound-producing mechanism, it is probable that they would produce similar calls^37^. The specific coding of sounds is likely a result of a combination of both the morphology of the sound-producing mechanism^28,38,39^ and the neurophysiology associated with sound production^40^. This suggests that variations in acoustic signals stem from both structural and functional aspects of sound generation.

**Fig. 1:**
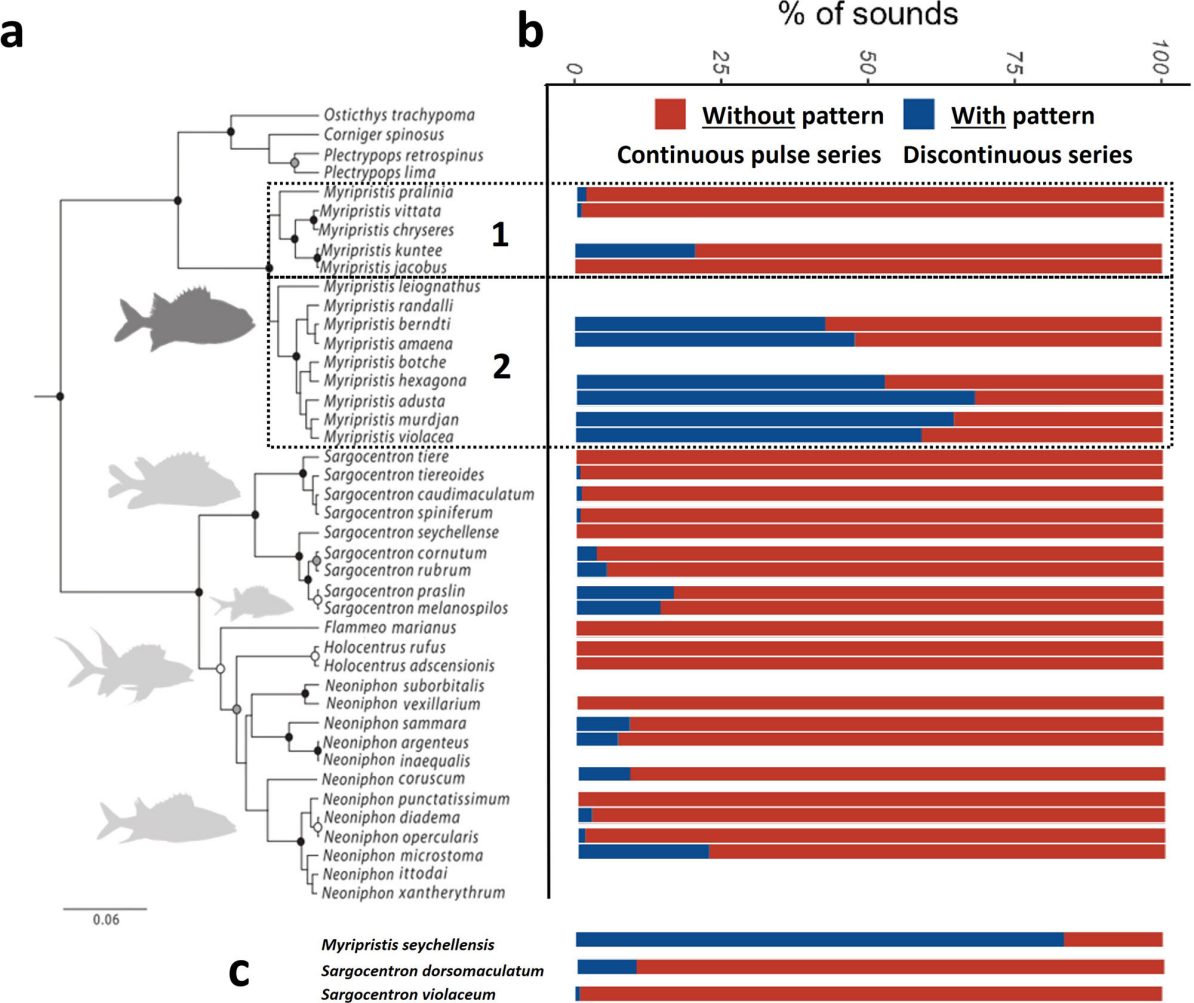
Relationships between the holocentrid species ability to produce sounds with pattern and their positioning in the most recent phylogeny of this family. **a** Phylogenetic tree of Holocentridae modified from Dornburg et al.^44^ and **b** corresponding histogram of the percentage of sounds consisting in discontinuous series of pulses (i.e., made of several blocks of pulses or pattern) and in continuous series of pulses (i.e., made of one block of pulses or without pattern). The numbers 1 and 2 refer to the two subgroups within the genus *Myripristis*. **c** Species that are not found in the existing phylogenety. Due to its high percentage of sounds with pattern (formed by blocks of pulses), *Myripristis seychellensis* was considered as belonging to species of group 2. *Sargocentron violaceum* seems to be closely related to *Sargocentron spiniferum* and *Sargocentron caudimaculatum*^45^. The position of *Sargocentron dorsomaculatum* in the tree is unknown.

Fishes of the family Holocentridae (Holocentriformes) are vocal species that occur in marine, tropical, and subtropical reef environments worldwide^41,42^. Based on the morphology of the swim bladder shape and auditory bulla, holocentrids have been divided into two subfamilies: Myripristinae (soldierfishes) and Holocentrinae (squirrelfishes^43^). These two subfamilies are composed of 5 (*Corniger, Pristilepis, Plectrypops, Ostichthys, Myripristis*) and 4 (*Holocentrus, Flammeo, Sargocentron, Neoniphon*) genera (Fig. 1a), respectively^44,45^. Among the 91 valid species of the family, 14 species from 4 genera are known to be vocal^12,39,46–50^. Those species were recorded either in the wild or under laboratory conditions. The sound-production mechanism of holocentrids relies on the contraction of paired bilateral sonic muscles originating on the skull and inserted on articulated ribs in tight connection with the swim bladder, causing its vibration, which produces sounds^39,46,51^. In *Holocentrus rufus*, Gainer et al.^52^ showed that the contraction rate of the sonic muscles determines the fundamental frequency (ca. 75–85 Hz). The general mechanism is consistent across four investigated holocentrid genera (*Holocentrus*, *Neoniphon*, *Sargocentron*, *Myripristis*), although morphological differences in muscle insertions and the number of ribs involved in the mechanism have been observed between these genera that could potentially explain differences in acoustic features^39^. Therefore, we expect sounds of closely related species (e.g., within a genus) to be more similar than sounds of phylogenetically distant species (e.g., between genera). Holocentrids serve as an ideal model to test this hypothesis for several reasons: (1) all species which have been investigated so far are vocal, (2) interspecific variations both in sounds and in the sound-producing mechanism have been reported^39^, (3) this family, composed of 2 subfamilies, 9 genera and 91 species, can be investigated at many taxonomic levels, (4) holocentrids are nocturnal fishes and could therefore rely significantly on sound production for communication^11^ and (5) often, closely related species are found living not only in sympatry but also in the same communities (i.e., the same habitats, such as caves), which requires species-specific acoustic signals for effective communication.

This study aims to determine the discriminability of the sounds produced by the holocentrids at different taxonomic levels: (1) subfamily, (2) genus, (3) the two main phylogenetic branches of the genus *Myripristis* (Fig. 1a), and (4) species. Each of the different taxonomic levels was investigated, from the highest to the lowest, to seek whether sounds could be used as taxonomic indicators. This term refers to a specific characteristic or trait used to classify organisms into distinct taxonomic categories (e.g., species, genera, families, etc.). These indicators can include different kinds of features, which help systematically categorize and identify biological diversity based on evolutionary relationships.

This study represents the first large-scale investigation of fish acoustics (i.e., involving a high number of species). All recordings were made directly in the field, thus avoiding the distortion of the acoustical features that occurs when sounds are recorded in tanks^50^. Sounds were recorded while the fishes were hand-held. The advantage of this approach is that all specimens were recorded in standardized conditions (recorded in the same behavioral context and at the same distance from the hydrophone), which allows for reliable sound comparisons between taxa^27,29,53^.

Our methodology was conducted in two phases. Initially, we performed a detailed analysis of over 7600 sounds from 388 specimens to accurately determine the acoustic signature of each species. These data were then used to test whether the sounds could facilitate discrimination at various taxonomic levels. Specifically, we examined whether sounds from the same taxa could be grouped together and whether it is feasible to rely on sounds for taxon identification across different taxonomic ranks.

## Results

**Fig. 2:**
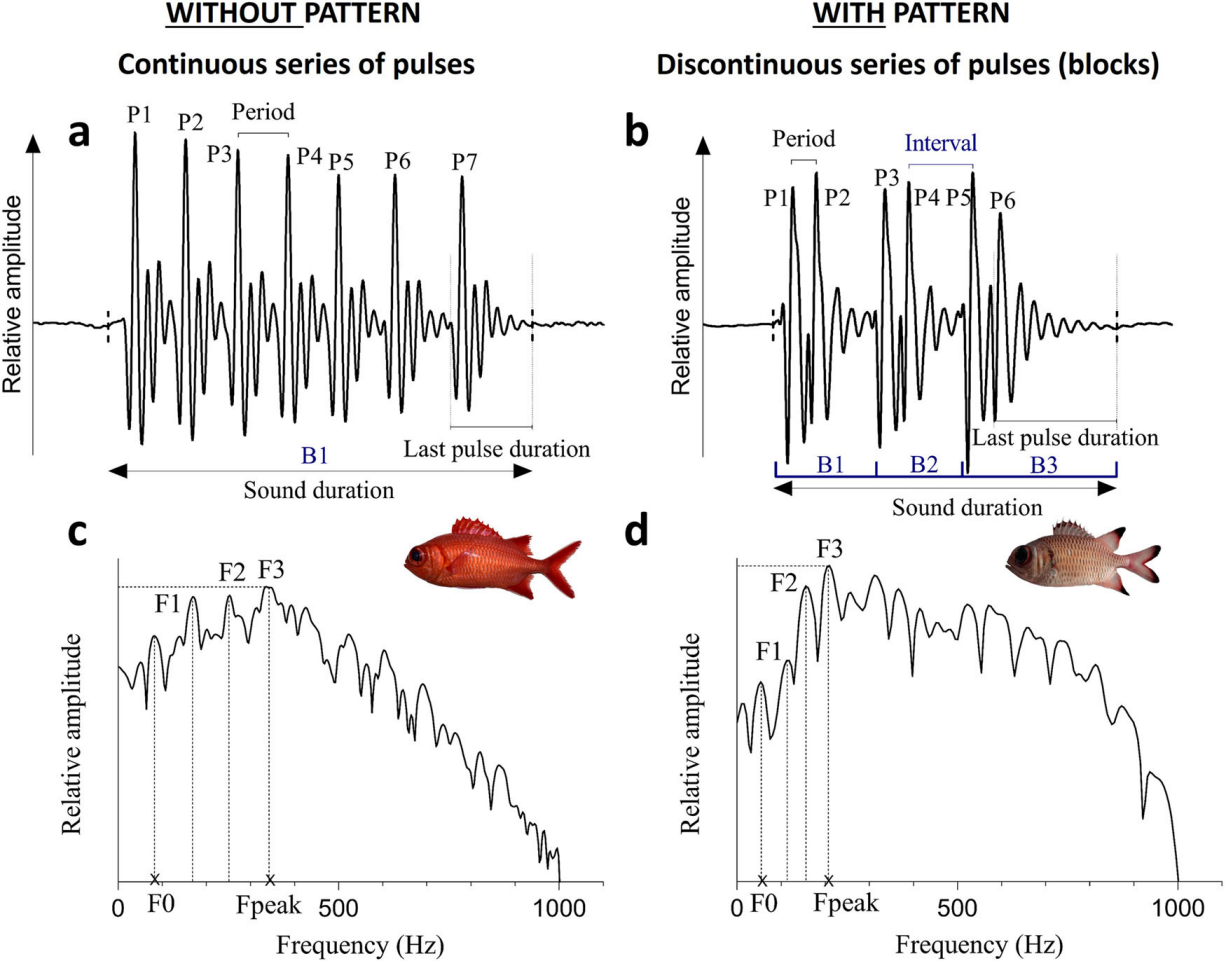
Illustration of the different acoustic features describing sounds. Oscillograms (**a**, **b**) with corresponding power spectra in *Myripristis vittata* and *M. adusta* (**c**, **d**), respectively. This comparison enables to highlight the difference between sounds without pattern that consist of a continuous series of pulses forming a single block (illustrated sound of *M. vittata* is composed of 7 pulses grouped in one block) and sounds with pattern consisting of a discontinuous series of pulses forming several blocks (illustrated sound of *M. adusta* is composed of three blocks made of two pulses each). P pulses; B blocks; F harmonics; F0 fundamental frequency; Fpeak dominant frequency.

All holocentrid species recorded share some common characteristics. The calls were composed of a variable number of pulses and, therefore, varied in duration. Sounds that were made of more than two pulses could possess harmonics, and their fundamental frequency never exceeded 220 Hz. Some of these sounds consisted in a continuous series of pulses and were therefore considered as deprived of the pattern (Fig. 2a). Other sounds consisted in a discontinuous series of pulses where pulses were grouped into several blocks; those sounds will be referred to as sounds with pattern (Fig. 2b). These sounds made of several blocks of pulses (with pattern) were mainly produced by *Myripristis* species of group 2 (Fig. 1a, b), whereas the other species mostly produced sounds without pattern (Fig. 2a). All descriptive statistics regarding acoustical variables will be presented as means ± standard deviation (sd) or minimum and maximum values. Descriptions of the sounds produced by each species are available in Supplementary Notes 1. An oscillogram of a representative sound of each species is also provided in Supplementary Fig. 1. In the results, “*n*” refers to the total number of analyzed sounds and “*N*” to the number of individuals, encompassing different species; *n* = y, *N* = *x* means that the analysis was made on y sounds coming from x specimens. From the 7662 recorded sounds, 779 sounds were made of 1 pulse (P1; *N* = 75, from 13 species), 639 of 2 pulses (P2; *N* = 104, from 20 species), 4635 of >2 pulses without blocks of pulses (P3; *N* = 334, from 32 species) while the 1609 remaining sounds were made of >3 pulses distributed in several blocks of pulses (P4; *N* = 186, from 25 species). Together, there were 6244 sounds made of >2 pulses, and they belonged to 346 specimens from 32 species (Supplementary Table 1).

For the various taxonomic levels, the different acoustical variables describing sounds for each taxon are summarized in Supplementary Tables 2–5. All variable correlation plots can be found in Supplementary Fig. 2.

### Regressions between size and acoustical variables

Because significant relationships were found between total length (TL) and acoustical variables (sound duration, number of pulses in sounds, duration of the final pulse in sounds, and dominant frequency) for several species (Regressions: *p* < 0.05; Supplementary Tables 6–9), these variables were divided, for the subsequent analyses, by total body length, following the formula “*X*(TL)^−1”^, where “*X*” is the acoustical variable. This allowed appropriate interspecific comparison by reducing the effect of fish size on acoustic variability^23,28^.

### Discriminability of the holocentrid sounds

At the highest taxonomic level, significant differences between sounds of the two subfamilies (Myripristinae and Holocentrinae), were observed. Sounds produced by Myripristinae were significantly longer (*t* test: t = −13.91, *df* = 378, *p* < 0.001) than sounds produced by Holocentrinae (74 ± 18 ms and 50 ± 18 ms, respectively; Supplementary Table 2), most probably because the number of pulses composing Myripristinae sounds (6.4 ± 1.7) was significantly higher (Wilcoxon–Mann–Whitney test: *W* = 5629, *p* < 0.001) than in Holocentrinae sounds (3.7 ± 1.9). The fundamental frequency of sounds was also significantly higher (Wilcoxon–Mann–Whitney test: *W* = 9731, *p* < 0.001) in the former group (115 ± 25 Hz) than in the latter one (90 ± 31 Hz). Since the period is inversely proportional to the frequency, it was also significantly smaller (Wilcoxon–Mann–Whitney test: *W* = 23966, *p* < 0.001) in Myripristinae (9.5 ± 2.7 ms) compared to Holocentrinae (12.7 ± 6.1 ms). Another main difference between these two groups was the larger presence of sounds with pattern (Wilcoxon–Mann–Whitney test: *W* = 8584, *p* < 0.001) in Myripristinae (41 ± 37%) compared to Holocentrinae (4 ± 9%). However, sounds from the two subfamilies did not diverge in terms of dominant frequency nor in the duration of their last pulse (Wilcoxon–Mann–Whitney test: respectively, *W* = 19349 and 15256, *p* > 0.05).

At the genus level, all acoustical variables, except the interval, were significantly different between genera (Kruskal–Wallis: *p* < 0.05; Supplementary Table 10). Particularly, *Myripristis* sounds were longer (73.9 ± 17.9 ms), composed of more pulses (6.4 ± 1.7) and blocks of pulses (1.6 ± 0.7) than sounds of all Holocentrinae genera (*Flammeo*: 18.1 ± 2.2 ms, 1.2 ± 0.1 pulses, 1 ± 0 block; *Holocentrus*: 41.3 ± 11 ms, 4.3 ± 0.6 pulses, 1 ± 0 block; *Sargocentron*: 56.8 ± 11.2 ms, 3.5 ± 1.3 pulses, 1 ± 0.1 blocks; *Neoniphon*: 45.1 ± 21.6 ms, 4.1 ± 2.5 pulses, 1.1 ± 0.1 blocks, respectively) (Dunn’s tests: *p* < 0.025; Supplementary Table 3; Supplementary Table 11). Besides, *Myripristis* sounds diverged from *Sargocentron* sounds (72 ± 25 Hz, 15.6 ± 6.9 ms) in having a higher fundamental frequency and lower period (115 ± 25 Hz, 9.5 ± 2.7 ms), from *Neoniphon* sounds (15.6 ± 5.8 ms) in having their last pulse of longer duration (17.3 ± 6.4 ms) and from both genera sounds (285 ± 63 Hz and 402 ± 113 Hz, respectively) in having a lower dominant frequency (260 ± 58 Hz) (Dunn’s tests: *p* < 0.025; Supplementary Table 11). Some differences were also found between genera within Holocentrinae (Kruskal-Wallis: *p* < 0.05; Supplementary Table 10). Sounds produced by *Flammeo marianus* were shorter and composed of a lower number of pulses (Dunn’s tests: *p* < 0.025; Supplementary Table 11) than *Neoniphon* sounds, themselves composed of a higher number of pulses and having a higher dominant frequency than *Sargocentron* sounds (Dunn’s tests: *p* < 0.025; Supplementary Table 11). *Sargocentron* sounds were distinguished from *Neoniphon* and *Holocentrus* sounds by also having a lower fundamental frequency and a larger period (Dunn’s tests: *p* < 0.025; Supplementary Table 11). Finally, the final pulse of *Holocentrus* sounds was also shorter than that of both *Neoniphon* and *Sargocentron* sounds (Dunn’s tests: *p* < 0.025; Supplementary Table 11).

Given that the sounds of *Myripristis* significantly differed from those of other genera and because they were not part of the same subfamily, the analysis was specifically repeated for Holocentrinae genera only. This approach was taken to ensure that the distinct characteristics of *Myripristis* sounds did not obscure potential differences among these genera. For this analysis, we excluded the variables related to pattern, since those mainly concerned *Myripristis* sounds. This second analysis showed additional acoustical differences between Holocentrinae genera sounds (Kruskal–Wallis: *p* < 0.05; Supplementary Table 12). *Holocentrus* sounds were shorter than the sounds of *Sargocentron,* and sounds produced by *Flammeo* were made of a lower number of pulses than these two genera (Dunn’s tests: *p* < 0.025; Supplementary Table 13).

**Fig. 3:**
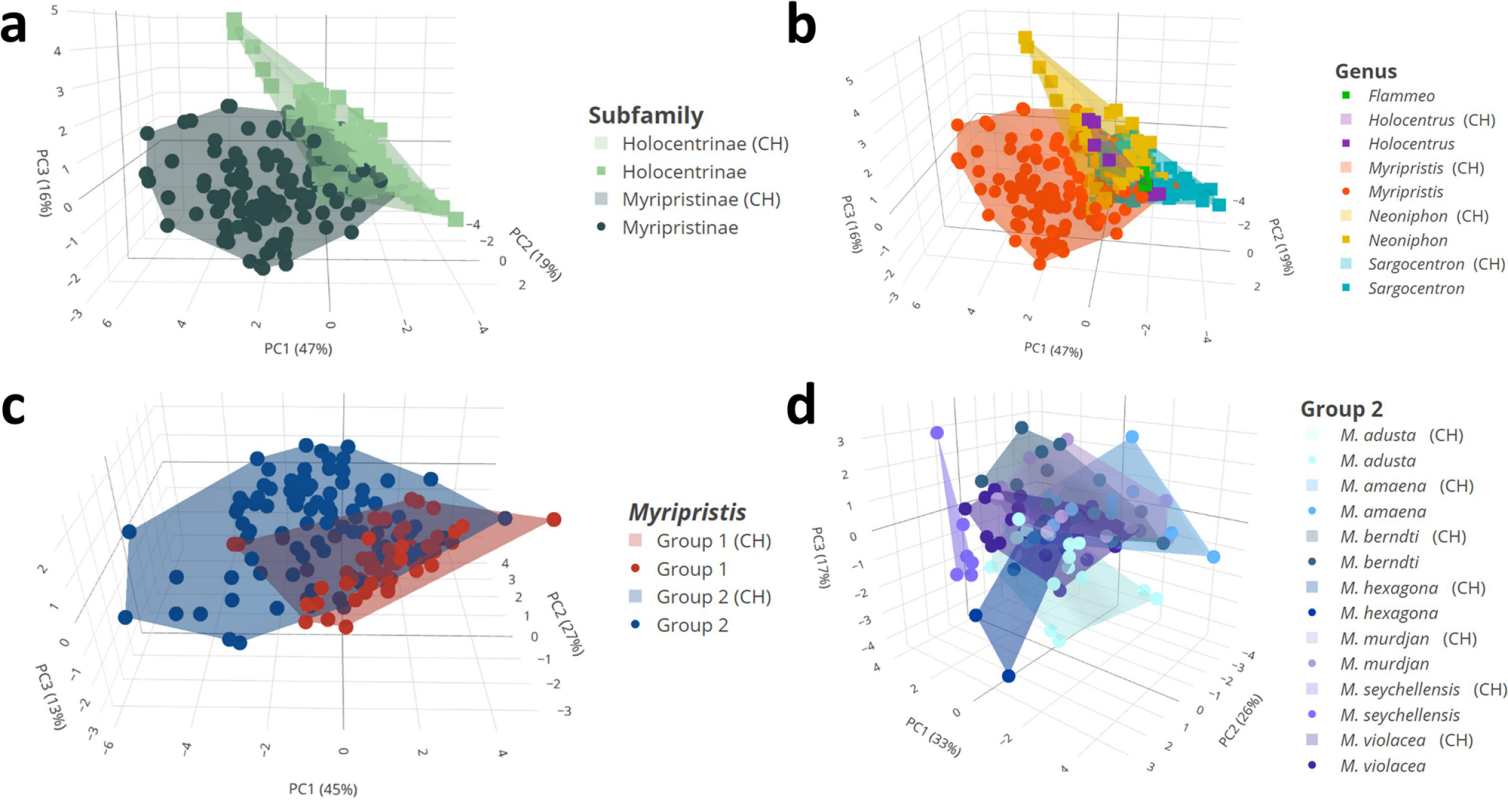
Scatterplots of the first three principal components (PC1, PC2, PC3) based on various acoustic features in Holocentridae across different taxonomic levels. Scatterplots of the sounds (*N* = 365) at the subfamily level (**a**) and genus level (five genera) (**b**) were performed with six acoustical variables (sound duration, number of pulses, fundamental and dominant frequencies, pulse period, and duration of the last pulse). Scatterplot of the sounds at the subgeneric level **c** illustrated by the two groups of *Myripristis* (*N* = 176) was performed with eight acoustical variables (sound duration, number of pulses, fundamental and dominant frequencies, pulse period and duration of the last pulse, number of blocks in sounds, percentage of sounds with pattern). Scatterplot of the sounds at the species level (**d**) illustrated by *Myripristis* species of group 2 (*N* = 101) was performed with 10 acoustical variables (sound duration, number of pulses, fundamental and dominant frequencies, pulse period, and duration of the last pulse, number of blocks in sounds, number of pulses in blocks, interval, percentage of sounds with pattern). CH convex hull. 3D scatterplots are available in Supplementary Videos 1-4.

**Fig. 4:**
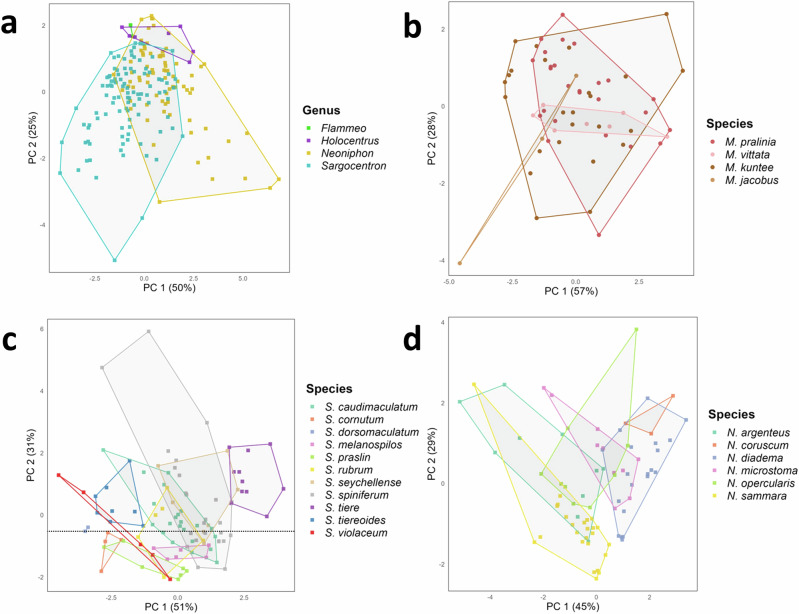
Scatterplots of the first two principal components (PC1 and PC2) based on various acoustic features in Holocentridae across different taxonomic levels. All scatterplots were performed with six acoustical variables (sound duration, number of pulses, fundamental and dominant frequencies, pulse period, and duration of the last pulse) of the sounds produced by (**a**) the different genera of Holocentrinae (*N* = 189), **b** *Myripristis* species of group 1 (*N* = 61), **c** *Sargocentron* species (*N* = 101), **d** *Neoniphon* species (*N* = 77).

Results of the principal component analysis (PCA) at the subfamily and genera levels showed, in agreement with the univariate statistical analyses, that although there was a small overlap, sounds of both the two subfamilies and the five genera differentiate well (Fig. 3a, b; Supplementary Videos 1–2). The first three principal components (PCs) accounted for 47, 19, and 16% of the variability, respectively, cumulatively accounting for 83% of the total variation. The sound duration, number of pulses in sounds, fundamental frequency, pulse period, percentage of sounds with pattern, and number of blocks in sounds mostly contributed to PC1, whereas the duration of the final pulse in sounds and dominant frequency were principally associated with, respectively, PC2 and PC3. When considering Holocentrinae genera only, PC1 and PC2 explain 50 and 25% of the acoustical variation, respectively, with a cumulative explained variation of 75% (Fig. 4a). In this case, the sound duration, number of pulses in sounds, fundamental and dominant frequencies and pulse period mainly contributed to PC1, whereas the duration of the final pulse in sounds was mostly associated with PC2. Whether the convex hull (CH) volumes were adjusted for the number of species (RCHS) or for the number of specimens (RCHI), the analysis revealed that Myripristinae (RCHS: 10.96 and RCHI: 0.68) utilizes an acoustic space nearly three times greater than Holocentrinae (RCHS: 2.4 and RCHI: 0.25) despite a lower number of species and specimens sampled for that group (Supplementary Table 14). In Holocentrinae, *Neoniphon* occupied the largest acoustic space proportionally to the number of species and specimens sampled, followed by *Sargocentron* and then *Holocentrus* (Supplementary Table 14).

The discriminative ability of acoustical variables to distinguish species within the family Holocentridae varied across the different taxonomic levels. Based on the acoustical variables, the flexible discriminant analysis (FDA) calculated an overall correct classification rate (CCR) of 88%, with 322 out of the 365 recorded individuals (IDs) were correctly classified at the subfamily level. At the genus level, when considering all genera combined, the overall CCR was 81% (296/365), with individuals from the genus *Myripristis* being correctly classified at 91.5%. The overall CCR was also 81% (155/189) at the genus level when considering only Holocentrinae genera. The CCRs vary according to the genus; *Sargocentron* (85.4%) and *Neoniphon* (83.1%) were largely correctly classified. In contrast, the categorization accuracy was only 42.9% in *Holocentrus* and 0% in *Flammeo*. The small sample sizes of these last genera (seven and two individuals, respectively) could contribute to their low CCR values.

The differentiation was even more challenging at the species level when all species were considered together. In this case, the overall CCR was only 50% (184 out of 365), which means that only half of the species can be correctly attributed based on their acoustic signatures. This low accuracy at the species level indicated overlap in acoustic characteristics among species within the genera, demonstrating the challenges of using acoustical variables alone for species-level identification in Holocentridae. The following results, however, showed that CCR increased when species were compared within the same generic group.

Species of the first group mostly produced sounds that rarely exhibited a pattern (9 ± 19%; Figs. 1b, 2a) while species of the second group produced many sounds that did show a pattern (57 ± 34%; Figs. 1b, 2b) (Wilcoxon–Mann–Whitney: W = 888, *p* < 0.001) (Supplementary Tables 4, 15). This was also emphasized by a smaller number of blocks of pulses in the sounds of group 1 (1.1 ± 0.3) with respect to sounds of group 2 (1.9 ± 0.7) (Wilcoxon–Mann–Whitney: W = 880, *p* < 0.001; Supplementary Table 4). In the rare cases where group 1 species produced sounds with pattern, these sounds had typically longer intervals (19.9 ± 3.7 ms) than in the sounds of group 2 (16.1 ± 3.8 ms) (Wilcoxon–Mann–Whitney: W = 1432, *p* < 0.001). For a same mean duration of about 70-75 ms (Wilcoxon–Mann–Whitney: W = 3285, *p* > 0.05), sounds of group 2 were made of more pulses than sounds of group 1 (Wilcoxon–Mann–Whitney: W = 2796, *p* < 0.05). This in accordance with a shorter pulse period in group 2 (8.5 ± 2.1 ms) than in group 1 (11.5 ± 2.6 ms), which also corresponded to a higher fundamental frequency of the sounds (Wilcoxon–Mann–Whitney: W = 6176 & 914, *p* < 0.001). No difference was observed in terms of dominant frequency nor in the duration of the last pulse (Wilcoxon–Mann–Whitney: W = 3864 & 3866, *p* > 0.05).

Results of PCA similarly showed that the sounds of the two groups of *Myripristis* diverged from one another (Fig. 3c; Supplementary Video 3). The first three PCs respectively accounted for 45, 27 and 13% of the variability, for a cumulative explained variation of 85%. While the duration of the last pulse was mainly associated with both PC2 and PC3, all the other variables mainly contributed to PC1. The relative CHs volumes calculated for each group of *Myripristis* indicated that *Myripristis* of group 2 occupied more than twice as much acoustical space than *Myripristis* of group 1 (17.4, 1.06 and 8.6, 0.56, respectively).

**Fig. 5:**
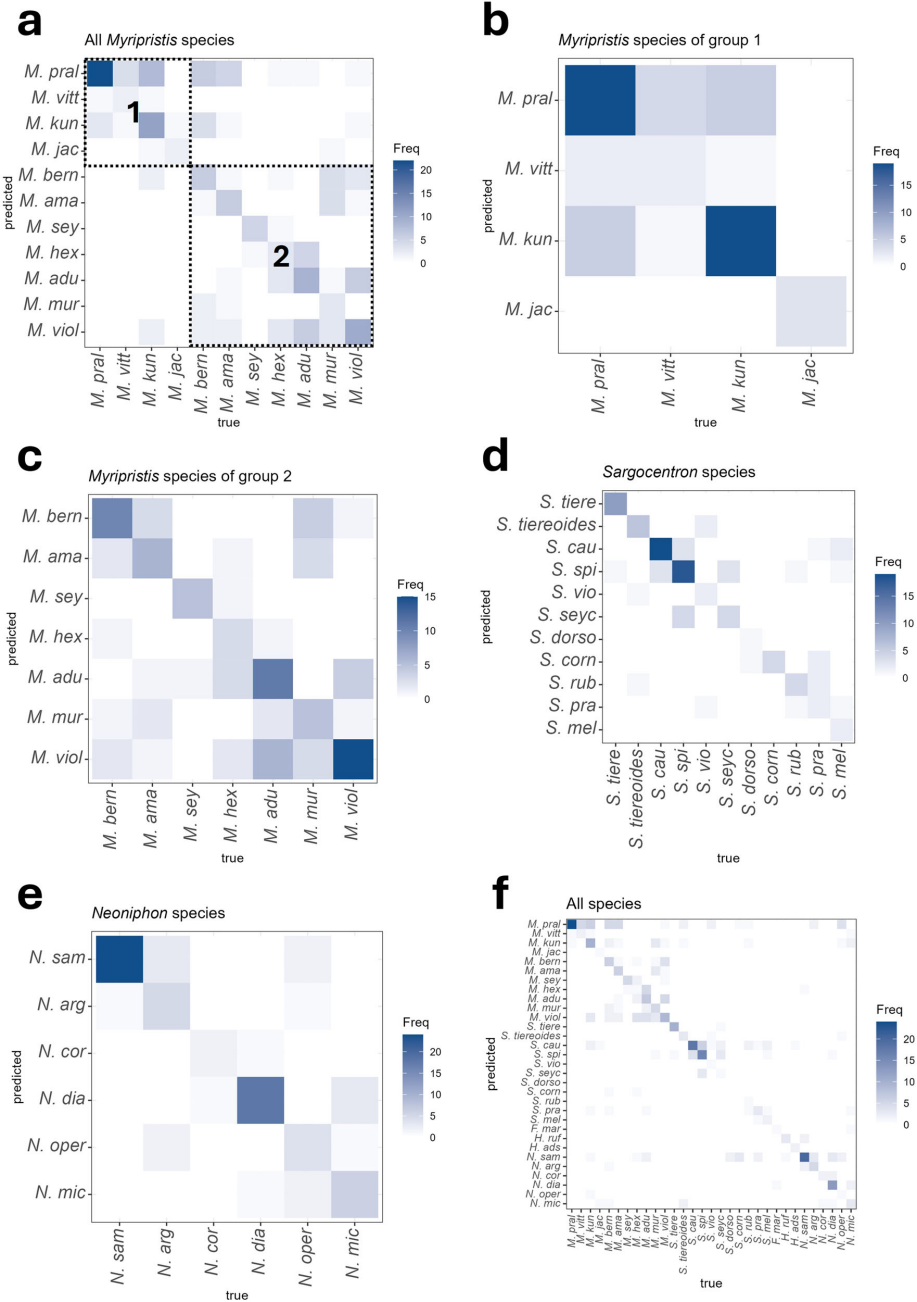
Confusion matrices showing the conditional frequency of classification of holocentrids based on sounds at different taxonomic levels resulting from flexible discriminant analyses. Matrices concern (**a**) *Myripristis* species, **b** *Myripristis* species of group 1, **c** *Myripristis* species of group 2, **d** *Sargocentron* species, **e** *Neoniphon* species, and (**f**) all species of Holocentridae investigated. Variables used to perform these analyses were those used in the PCAs. The probability of correct classification is found in the diagonal of the matrix. Boxes in (**a**) indicate probabilities of classification that correspond to the same *Myripristis* group.

The FDA indicated that 88% (155/176) of the recorded specimens were correctly classified as belonging to their respective *Myripristis* group. When considering the lower taxonomic levels (i.e., species of both groups combined), the CCR was however only 45% (79/176), which indicated that more than half of the individuals would be correctly classified as belonging to the right species based on sounds. The confusion matrix (Fig. 5a), which has been ordered based on their position in the holocentrid phylogenetic tree (Fig. 1a), indicated that misclassification errors mainly occurred among species within each group.

Univariate statistical analyses carried out on acoustical variables of sounds of species of *Myripristis* of group 1 revealed only one difference in fundamental frequency between *M. jacobus* and *M. pralinia* sounds (ANOVA: *df* = 3, F = 3.18, *p* < 0.05; Tukey’s test: *p* < 0.05; Supplementary Tables 5, 16, 17). Similarly, results of the PCA indicated that species of this group can hardly be identified based on their sounds, as observed by the overlapping of the four species, especially *M. pralinia* and *M. kuntee* (Fig. 4b). The PC1 and PC2 accounted, respectively, for 57 and 28% of the variability, for a total explained variance of 85%. All variables mostly contributed to PC1, except the pulse period which was mainly associated with PC2. The PCA did not allow a clear distinction of the different species due to much overlap, although *M. jacobus* differentiated a bit from the other species. *Myripristis vittata* had less variability in its sounds with respect to the other species. The FDA gave a CCR of 70% and could classify 43 individuals out of 61 into the correct species. The CCRs varied according to the species of the group (Fig. 5b): the categorization accuracy exceeded 70% in both *M. kuntee* and *M. pralinia* and reached 100% in *M. jacobus*. However, it was only 28.6% in *M. vittata*.

Several acoustical variables (sound duration, number of pulses, fundamental and dominant frequencies, last pulse duration, pulse period, interval, and number of pulses in blocks) differed significantly between *Myripristis* species of group 2 (ANOVA and Kruskal–Wallis tests: *p* < 0.05; Supplementary Tables 18-19). For instance, one general tendency was the significantly longer interval between blocks of pulses (23.8 ± 3 ms) and lower number of pulses composing each block (1.9 ± 0.1) in *M. seychellensis* sounds in contrast to the sounds of all other species within this group (Dunn’s tests: *p* < 0.025; Supplementary Tables 5, 20). Moreover, sounds of *M. seychellensis* were generally shorter, composed of fewer pulses, had a fundamental frequency significantly larger and a dominant frequency significantly smaller than most species (i.e., *M. amaena, M. berndti* and *M. murdjan*) (Dunn’s & Tukey’s tests: *p* < 0.025; Supplementary Tables 20-21). More broadly, fundamental frequency appeared to be a differentiating factor in the sounds of many pairs of species (Dunn’s tests: *p* < 0.025; Supplementary Table 20).

The first three PC respectively explained 33, 26 and 17% of the variance, for a total of 76%. The sound duration, number of pulses in sounds, dominant frequency, and number of blocks in sounds mostly contributed to PC1, whereas the interval, the number of pulses in blocks, and the percentage of sounds with pattern were mainly associated with PC2. Finally, the fundamental frequency, the duration of the final pulse and the pulse period were mainly associated with PC3. In agreement with the results of univariate analyses, the PCA indicated that sounds of *M. seychellensis* differentiate quite well from the sounds of the other species, except for *M. hexagona* (Fig. 3d; Supplementary Video 4). Similarly, the sounds of *M. adusta* also differentiated this species from the other species. However, there was still a lot of overlapping between species of this group. Overall CCR for species of this group was 46%, with 55 individuals out of 101 correctly classified as belonging to the right species. CCRs varied according to the species (Fig. 5c). As observed from the PCA, the categorization accuracy of *M. seychellensis* was quite high (83.3%). It then decreased for *M. violacea* (71.4%), *M. berndti* (62.5%) and *M. adusta* (55%) to less than 50% in *M. amaena, M. hexagona* and *M. murdjan*.

Univariate statistical analyses revealed differences in all acoustical parameters between the different *Sargocentron* species (Kruskal–Wallis tests: *p* < 0.01; Supplementary Table 22). Importantly, sounds of *S. tiere* had a pulse period 2 to 3 times longer (32 ± 6.7 ms) and a fundamental frequency shorter (33 ± 5) than the sounds of all the other species (Dunn’s tests: *p* < 0.025; Supplementary Tables 5, 23). They also differentiated from all species by their low number of pulses (1.9 ± 0.5), except for *S. seychellense* whose sounds were also composed of a reduced number of pulses (1.9 ± 0.4). Some acoustical variables of sounds of *S. tiereoides* also differed significantly from many species (Dunn’s tests: *p* < 0.025; Supplementary Table 23). They were significantly longer (61 ± 9 ms) than the sounds of *S. tiere* (55.3 ± 17.1), *S. spiniferum* (55.1 ± 10.1 ms) and *S. seychellense* (44.4 ± 5.9 ms), but shorter than the sounds of *S. melanospilos* (51.1 ± 11 ms) and *S. praslin* (56.3 ± 4.3 ms). Sounds of *S. tiereoides* were also composed of significantly more pulses (4.7 ± 0.6), had a higher fundamental frequency (93 ± 6 Hz) and shorter pulse period (11.3 ± 1 ms) than *S. tiere* (1.9 ± 0.5 pulses; 33 ± 5 Hz; 32 ± 6.7 ms)*, S. caudimaculatum* (3.8 ± 0.7 pulses; 66 ± 6 Hz; 14.6 ± 1.7 ms)*, S. spiniferum* (2.7 ± 0.7 pulses; 57 ± 10 Hz; 16.7 ± 3 ms) and *S. seychellense* (1.9 ± 0.4 pulses; 62 ± 17 Hz; 16.9 ± 5.3 ms) (Dunn’s tests: *p* < 0.025; Supplementary Table 23). The dominant frequency of *S. tiereoides* sounds was also higher (384 ± 119 Hz) than that of *S. tiere* (256 ± 32 Hz)*, S. caudimaculatum* (272 ± 50 Hz)*, S. spiniferum* (244 ± 30 Hz) and *S. praslin* (291 ± 29 Hz). Similarly, sounds of *S. violaceum* were composed of more pulses (5.2 ± 0.6) than those of *S. tiere, S. spiniferum* and *S. seychellense* (Dunn’s tests: *p* < 0.025; Supplementary Table 23) and had a higher fundamental frequency (95 ± 8 Hz) and lower pulse period (11 ± 0.9 Hz) than the latter three species and also *S. caudimaculatum* (Dunn’s tests: *p* < 0.025; Supplementary Table 23). Several additional differences could be observed between the sounds of *S. seychellense* and *S. spiniferum* and the sounds of *S. cornutum, S. rubrum, S. praslin, S. melanospilos, S. dorsomaculatum*. No difference was however observed between species within these two sets of species and there were only two differences in the acoustical variables of the sounds produced by *S. dorsomaculatum* and the rest of the species (Dunn’s tests: *p* < 0.025; Supplementary Table 23).

The PC1 and PC2 explained, respectively, 51 and 31% of the acoustical variation, for a total explained variation of 82%. The number of pulses in sounds, the fundamental and dominant frequencies and the pulse period mostly contributed to PC1, whereas the duration of the final pulse mainly contributed to PC2. Sound duration was greatly associated with both axes. The upper part of the PCA was mainly occupied by *S. tiere, S. tiereoides, S. caudimaculatum, S. spiniferum* and *S. seychellense*, while the lower part was mostly occupied by *S. cornutum, S. praslin* and *S. melanospilos. Sargocentron rubrum* distribution spread to both the upper and lower parts of the PCA*. Sargocentron tiereoides* also occupied an area quite apart from all species, especially from *S. caudimaculatum, S. spiniferum, S. seychellense* and *S. tiere* (Fig. 4c). As expected from the results of univariate statistical analyses, sounds of *S. tiere* were located in a region distinct from the other species within the PCA. *Sargocentron spiniferum* and *S. seychellense* overlapped almost completely, which corresponded to the results of the univariate analyses. Overall CCR for *Sargocentron* species was 70%. The FDA could correctly classify 72 individuals out of 103 as belonging to the right species. Again, the CCRs varied according to the species (Fig. 5d): *S. cornutum* (100%)*, S. tiere* (90.9%), *S. caudimaculatum* (86.4%), *S. tiereoides* (75%) and *S. spiniferum* (72%) were largely well classified by the FDA, while *S. violaceum* (40%), *S. melanospilos* (33.3%) and *S. praslin* (28.6%) were mostly incorrectly classified. Finally, the CCRs of *S. rubrum* and *S. seychellense* were 66.7% and 57.1%, respectively.

**Fig. 6:**
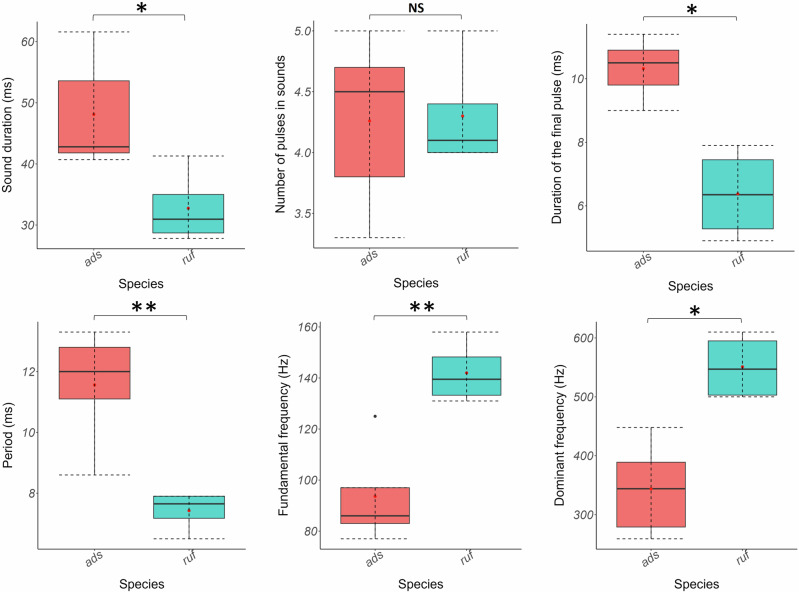
Boxplots of the acoustical variables of the sounds of the two *Holocentrus* species. *p < 0.05; **p < 0.01; ***p < 0.001. NS nonsignificant.

All acoustical parameters, except the number of pulses (Wilcoxon–Mann–Whitney: *W* = 0, *p* > 0.05), allowed the differentiation of the sounds of the two *Holocentrus* species (*t* tests & Wilcoxon–Mann–Whitney tests: *p* < 0.05; Fig. 6; Supplementary Table 5). *Holocentrus adscensionis* sounds were longer, featuring extended pulse period and last pulse duration for a similar number of pulses, along with lower fundamental and dominant frequencies. Based on their sounds, all individuals could be discriminated against and associated with the right species using the discriminant analysis.

Univariate statistical analyses revealed several significant acoustical differences between *Neoniphon* species (ANOVA: *df* = 6, F = 31.85, *p* < 0.001 for Npulses; Kruskal-Wallis tests for the other variables: *p* < 0.05; Supplementary Table 24). Mainly, sounds of *N. sammara* differentiated from the sounds of the other species of the genus, except *N. microstoma*, in their duration (Dunn’s test: *p* < 0.025; Supplementary Tables 5 and 25). Lasting 51 ± 11.4 ms, they had an intermediate duration between *N. argenteus* sounds (55.6 ± 11.5 ms) and *N. opercularis* sounds (76.4 ± 9.7 ms) and the species with the shortest sounds (*N. coruscum*: 21.7 ± 10.6 ms*, N. diadema*: 27.2 ± 8.7 ms and *N. punctatissimum*: 21.9 ± 7.6 ms). They also differentiated from the latter three species by their number of pulses (5.4 ± 1.2 *vs* respectively, 1.6 ± 0.8, 1.6 ± 0.8 and 1.1 ± 0.1; Tukey’s test: *p* < 0.01; Supplementary Table 26). Sounds of *N. sammara* also had a higher fundamental frequency and shorter pulse period than all species, except *N. argenteus*, whose frequency was also higher than the sounds of *N. coruscum, N. diadema,* and *N. punctatissimum* (Dunn’s test: *p* < 0.025; Supplementary Table 25). Sounds of the latter species were also shorter and composed of less pulses than all other species (Tukey’s test, *p* < 0.01; Supplementary Table 26). Many differences in acoustical variables could be reported between the sounds of *N. argenteus* and the sounds of these three species, especially in duration, number of pulses, and fundamental frequency (Dunn’s & Tukey’s tests: *p* < 0.025; Supplementary Tables 25, 26).

The PC1 and PC2 explained, respectively, 45 and 29% of the acoustical variation, for a total explained variation of 74%. The sound duration, the number of pulses and the duration of the final pulse mainly contributed to PC1, whereas PC2 was mostly associated with the dominant frequency. Both the fundamental frequency and the pulse period were greatly associated with the two axes. Although there was some overlap between species, *N. sammara* distinguished well from all species except *N. argenteus*, in agreement with the results of the univariate analyses (Fig. 4d). The sounds of the two species were the most similar, as observed by their overlapping on the PCA. While *N. sammara* also differentiated from *N. opercularis*, it was not completely the case for *N. argenteus*. A second group formed by the overlapping of *N. coruscum, N. diadema* and *N. punctatissimum* distinguished itself from the first group formed by *N. sammara* and *N. argenteus*. The FDA calculated an overall CCR of 77% for *Neoniphon* species. In other words, it classified 59 individuals out of 77 as belonging to the right species based on their acoustical variables. Similar to *Sargocentron*, the CCRs varied according to the species (Fig. 5e) : *N. sammara* (96%) and *N. diadema* (90%) were largely correctly classified, whereas CCRs of the other species varied between 44.4 and 66.7%.

## Discussion

We aimed to assess the existence of a species-specific signature in hand-held sounds produced by holocentrids and to determine the discriminability of these sounds at different taxonomic levels. This study is the first to examine such a broad scale of species at different taxonomic levels and shows that sound can serve as an effective taxa discriminator at various taxonomic levels, with discriminability efficiency being directly correlated to taxonomic hierarchy: the higher the taxonomic levels, the better the discriminability. Because misclassifications frequently occur between closely related species, multimodal species identification is supported.

Historically, studies on the species-specific character of sounds aimed to ascertain whether these signals could serve as prezygotic barriers, preventing reproduction between individuals of different species^33,34^. From this perspective, our comparisons of hand-held sounds may appear less relevant, as these sounds are not produced in courtship contexts. However, since they are produced in the same behavioral context, indicating that they result from genetic characteristics, they could still encode species identity information, as can be the case for morphological features.

Our study indicates that acoustic discriminability of taxa improves at higher taxonomic levels. Higher taxonomic levels imply greater variations in the sound-producing mechanism between taxonomic groups, which may accentuate differences in acoustical signals. Conversely, variations in the sound-producing mechanism at lower taxonomic levels are less pronounced, correlating with a reduced ability to produce distinct sounds. We show significant differences between the two subfamilies of Holocentridae in sound duration, number of pulses, fundamental frequency, and pulse period but also in the percentage of sounds with pattern. Despite the sounds of Holocentrinae genera exhibiting greater similarity to each other compared to those of the genus *Myripristis*, the acoustic characteristics of their calls still allow their differentiation. Only a few studies have assessed the acoustical discriminability of taxa at different taxonomic levels within a large taxon. In cats of the family Felidae, the distribution of three close-range vocalization types is fully concordant with its phylogenetic tree^54^. In teleosts, a single study has shown that sounds can be representative of the genus level in piranhas^30^. Using a linear discriminant analysis, *Pygocentrus* and *Serrasalmus* genera can be discriminated with an overall CCR of 84.9%.

Moreover, the acoustic features of the genus *Myripristis* effectively enable the delineation of two distinct groups, which precisely match the distribution of *Myripristis* species in the phylogenetic tree of Holocentridae (Fig. 1a). While species of group 1 mainly produce sounds without pattern, species of group 2 produce many sounds with pattern. Similarly, in *Sargocentron*, the differences between sounds of the different species appear to generally correspond to the topology of the phylogenetic tree for this genus (Fig. 1a). Notably, *S. tiere*, *S. tiereoides, S. caudimaculatum, S. spiniferum* are found in the first branch while *S. cornutum, S. praslin* and *S. melanospilos* are found in the second branch. This grouping aligns with the distinctions in their sounds (Figs. 1b, 4c), suggesting a potential relationship between acoustic and genetic distances, although further research would be needed for confirmation. Similarly, *N. argenteus* and *N. sammara* are very closely related phylogenetically, and their sounds are the most similar within *Neoniphon* species (Figs. 1a, 4d). These findings suggest a phylogenetic signal on acoustical features of calls in Holocentridae.

These original differences most likely stem from two primary factors: the anatomy of the sound-producing apparatus and the neurophysiology associated with the mechanism. While the sonic mechanism of all holocentrid species is fundamentally similar, significant morphological variations potentially responsible for the production of distinct sounds have been observed between Myripristinae and Holocentrinae^39^. To go deeper into these distinctions, further comparisons at the genera and subgenera levels need to be undertaken to determine potential relationships between acoustic characteristics and fine morphology. Variations in the sounds produced by different species sharing a similar sound-producing mechanism could also be attributed to differences in neuronal activations that could drive divergence in the temporal domain. This neurophysiological plasticity can be employed to generate various types of sounds using the same mechanism in different situations, such as (1) adaptability to other species^55^ or (2) to distinguish different species^26,56^.

Interestingly, the differences observed in terms of relative volumes between the CHs of the two subfamilies, the different genera and the two *Myripristis* groups, indicate that some taxa may have a greater potential to adapt or enhance their acoustic features. This capability suggests their ability to effectively utilize and diversify within larger acoustic spaces in their environment.

The importance of temporal patterning in species identification and fish communication, in general, has been known for several years^57,58^. In damselfishes (Pomacentridae), the temporal pattern of sounds, or pulse period, was reported as the most important factor for species recognition^33^. Different species have introduced new kinds of sound using the temporal pattern in their evolutionary history. Within *Gobius* (Gobiidae) lineage, different sounds and their corresponding emitting species, can be distinguished based on temporal patterns. Drumming sounds comprise sequences of discrete pulses, while tonal sounds consist of more rapidly repeated pulses. The relative tonal-to-pulsatile nature of these sounds is the most significant distinguishing property among the species as the species producing tonal sounds clustered together^23,59,60^. Additionally, one species, *Padogobius bonelli*, is capable of emitting complex sounds that include both tonal and drumming elements, allowing its identification^61^. As in Gobiidae, holocentrids also use temporal patterning to create distinctly specific sounds. In our study, we not only report on this phenomenon but also demonstrate that these fishes have taken a step further by displaying a unique complexity in sound patterns characterized by the temporal arrangement of pulses into distinct blocks resulting in the creation of novel sound varieties. The highest percentage of pattern in sounds was observed in group 2 of the genus *Myripristis* (>43%) with *M. adusta, M. amaena, M. berndti, M. hexagona*, *M. murdjan, M. seychellensis* and *M. violacea*, whereas group 1 with *M. jacobus, M. kuntee*, *M. pralinia* and *M. vittata* produced less than 2% of sounds with a pattern, except *M. kuntee* (20%.) Interestingly this difference corresponds to the division of the genus *Myripristis* into two distinct clades in the phylogeny of Holocentridae^44^. This special feature could have been selected during the speciation process as it provides the caller an additional way to be discriminated from sympatric species.

In teleosts, while an increasing number of studies have investigated and highlighted the species-specific character of sounds^23–30^, this concerns only a limited number of species. In the search for specific acoustic signatures, two scenarios can be considered. From an ethological perspective, it can be crucial for sympatric, closely related species to have clear distinctions in their calls to avoid confusion in communication and to ensure accurate species identification. This assertion is particularly relevant for fish, such as Holocentridae, that have pronounced nocturnal activity and cannot rely on visual communication. During daytime activities, other signals like color and movement may complement acoustic communication. Alternatively, from an evolutionary perspective, the sounds of closely related species are expected to share more common characteristics compared to those of phylogenetically distant species, as is generally the case with morphological traits. For this reason, analyses at the species level were conducted in two different ways: (1) comparing all holocentrid species and (2) comparing closely related species at a lower taxonomic level (i.e., within each genus).

In the comparison across taxa, the assessment of numerous species has generated divergent outcomes, emphasizing the notion that the expected species-specific character is not uniformly acquired. When investigating the species-specific character of sounds within each genus, the overall and species CRRs varied between genera. *Holocentrus* species were distinguishable with very high confidence (100%). Although differences existed between almost all species, only a few species of *Neoniphon* and *Sargocentron* could largely be discriminated from the others among each genus (e.g., *N. vexillarium* and *N. punctatissimum* due to their sounds composed of only one single pulse). *Myripristis* species could also hardly be discriminated against based on their sounds. These findings suggest that although sounds from holocentrids do contain species-specific information, there remains a considerable risk of misattributing a randomly selected sound to a species. Interestingly, misclassifications, however, do not seem to be random since most errors in classification occurred between closely related species (Fig. 5f). In other words, sounds of closely related species would be more similar, thus supporting the second scenario. The existence of a phylogenetic signal on acoustical properties has also been reported in studies using a high number of species. A large-scale study on drumming sounds of 92 species of woodpeckers reported acoustic character displacement only in rare cases where sympatric species were closely related^8^. In such cases, other cues (e.g., visual cues) could help species discrimination^8^. It is worth noting that the identity-signaling requirement for stress calls may not be as stringent as that for sounds produced during reproduction, potentially making them more useful for taxonomic differentiation at broader levels rather than at the species level.

One *Flammeo*, two *Holocentrus*, eight *Neoniphon*, 11 *Sargocentron,* and 11 *Myripristis* were investigated in this study. Discrimination among genera with fewer species was generally better. Moreover, FDA models applied to species by groups (among genera) performed better than when applied to the increasing number of species (all species combined). This observation leads to our second suggestion that the number of species considered in studies investigating the species-specific character of sounds in fishes would, at least partly, guide the discrimination results; the lower the number of species, the greater the discrimination based on sounds. Although most sounds are not as complex as those of higher vertebrates^62^, sound complexity could also affect species recognition. However, in bats, it has been similarly reported that while some species can be reliably identified from others based on echolocation calls, the task becomes challenging when considering a larger number of species or different study areas. This difficulty arises due to the overlap of acoustic features among certain species^1^.

Studies that investigated a higher number of species provide contrasting results that could be related to the kind of sounds. In European gobiids, Horvatić et al.^23^ used six acoustical variables to differentiate nine gobiid species belonging to five genera. Reporting a very high overall CRR of 92.5%, the authors suggested that the gobiid sounds were species-specific^23^. The relationship between sound variability and genetic distance was assessed, and it was reported that some acoustic features (sound duration, number of pulses, pulse repetition rate, and peak frequency) from representative sounds could carry, although quite weakly, a phylogenetic signal in vocal gobiids^23^. Similarly, Mélotte et al.^27^ reported that some piranhas species of *Serrasalmus* could be largely discriminated based on their sounds, but the overall CCR was only 53.6% for the eight closely related species considered using five acoustical parameters. A few years later, Raick et al.^30^ reported an overall CCR of 76.6% for 12 species of *Serrasalmus* and *Pygocentrus*. In contrast, although using eight acoustical variables, it has not been possible to discriminate between ten *Hypostomus* species^29^.

The divergence in these results may be attributed to the way the different fishes utilize their sounds; in other words, the information content of these sounds could vary across taxa in relation to their behavioral emission context. We would indeed expect sounds related to behaviors requiring conspecific recognition, such as reproduction, to be highly species-specific. Sounds used in behaviors that do not require species identification (e.g., agonistic interactions) do not need to be so specific^62^, which may imply a lower level of identity potential. Raick et al.^29^ proposed the same hypothesis due to the lack of differences between the acoustical features of catfishes of the genus *Hypostomus*. Sounds could, in this case, mainly have an anti-predatory function (alarm, distress, aposematism) since they are produced simultaneously to the erection of pectoral fins^29^. Sounds of the brown meager and the shi drum can be discriminated very efficiently using their acoustical features^24,63^. In Sciaenidae, vocalizations are known to play an important role in reproduction since male choice would be mainly based on visual and acoustical cues^64,65^. In Serrasalmidae, sounds are also species-specific, although they are not related to reproduction. In European gobiids producing very species-specific sounds, those were recorded during the reproductive season^23^. Like Scianidae, gobiids rely on these sounds for reproduction, which could explain their need for acoustical character displacement. In Holocentridae, sound production has been reported in different, mainly agonistic, contexts, such as territory defense, predator-signaling, mobbing behavior, chasing conspecific or heterospecific individuals, acoustically-mediated symbiotic relationships, etc.^12,14,18,46–49^.

The species-specific signature in sounds of Holocentridae is evident in some species but not in others, and this presents a wealth of information that is not easily discernible, as a study of such magnitude, encompassing such a large number of species, has never been conducted before. This study may challenge the assumption of species-specific signature in sounds, which has been erroneously accepted in the literature, as previous studies on this topic have typically focused on a small number of species. Moreover, due to the relative simplicity of many central and peripheral vocal mechanisms in fish (compared to tetrapods), they generally lack the ability to produce complex and dynamically frequency-modulated calls, thus limiting their capacity to produce various call types^66^. Similarly to morphological traits, natural selection does not uniformly affect a single type of character, suggesting that communication is not the sole driver for the emergence of new species. Speciation could occur while retaining common acoustic features. The fact that the sounds used in this study are not related to reproduction might also mask the existence of the ability to produce entirely species-specific sounds. However, even for reproductive purposes, the specific acoustic signature should not be considered an obligatory trait for at least two reasons: 1) reproductive communication is multimodal, and 2) constraints on species recognition are only required in sympatric species (which is not the case for all species studied here) sharing the same habitat. For instance, in damselfishes of the genus *Dascyllus*, acoustic character variation is more pronounced in *D. albisella*, the only member of the genus found in Hawaii and as such is subjected to fewer constraints compared to the three species living in sympatry in French Polynesia^26^.

This study investigating the species-specific character of sounds in fish has been performed through the recordings of the sounds produced by hand-held specimens, allowing the conduction of reliable comparisons between specimens and/or species because they are recorded in standardized conditions^27–29,50^. Although these sounds are produced in experimental contexts, they can provide significant information, such as indications of the type of sounds emitted by the recorded species in stressful situations. When species produce sounds while being held in hand, we can consider a mechanistic aspect where the produced sound is a reflective characteristic of the species. We hypothesize that hand-held sounds could be utilized in cladistics to aid in differentiating between closely related taxa, given their distinct acoustic signatures that reflect specific neurophysiological mechanisms. This approach may provide additional criteria for resolving phylogenetic relationships within clades where traditional morphological and genetic markers are inconclusive. This study, among others, demonstrates this particularly well, given the consistency of traits among specimens of the same species and the differences between species in the sounds produced by taxa in the same behavioral context. This method also offers a significant cost/benefit ratio compared to the resources required to record a similar number of sounds in natural conditions. Moreover, natural conditions also face criticism because internal and external environmental factors are not standardized, affecting comparisons. While the need for recognition among conspecifics in reproductive contexts is well understood, the use of calls in other behavioral contexts, such as agonistic interactions, may also vehiculate information to both conspecifics and heterospecifics. The acoustical signals themselves could increase the awareness of the whole community, regardless of the type of sound.

Our study shows, for the first time in teleosts, that sounds can be indicative of taxonomic grouping. They also indicate that taxa discriminability depends on taxonomic level; the higher the taxonomic level, the better the discrimination of taxa based on sounds. Moreover, a new kind of sound construction characterized by the existence of patterns in sounds with the distribution of several blocks of pulses was highlighted. Such complexity of call features may allow corresponding taxa to exploit unique acoustic spaces in their environment, as observed for *Myripristis* with respect to *Neoniphon, Sargocentron,* and *Holocentrus*. These results suggest a phylogenetic signal for acoustical features of calls in this family. We hypothesized that the morphology of the sound-producing apparatus would be responsible for differences in call features between taxa, as well as the neurophysiology associated with sound production. Finally, we suspect that the number of species considered in studies investigating the species-specific character of sounds would influence the results of the discrimination.

## Methods

### Data collection

In total, 388 holocentrid specimens from 2 subfamilies, 5 genera, 33 species (11 *Myripristis*, 11 *Sargocentron*, 8 *Neoniphon*, 2 *Holocentrus*, 1 *Flammeo*) belonging to 73 populations were collected between May 2019 and July 2022 by snorkeling or scuba diving in coral reef areas of Guadeloupe, French Polynesia, Guam, Seychelles and Philippines at a depth between 2 and 20 m (Supplementary Table 27). At night, individuals were caught with a hand net. During the day, a quinaldine solution was used to anaesthetize them for a few seconds, making it easier to catch them inside caves^48,67^. Sounds produced by fish were first recorded, and then the body size of each fish was measured, both in standard and total lengths, before being photographed (Supplementary Table 28). Measurements of size were missing for 23 individuals and were therefore excluded from the comparative analyses. As sexual dimorphism has never been reported in this fish family, the sex of the individuals was unknown.

### Sound recordings

The sounds of each specimen were recorded at sea. Water temperature during recordings ranged between 28 °C and 30 °C. Sounds were recorded using an HTI-96-min hydrophone (High Tech Inc., Long Beach, USA) (sensitivity: −164.4 dB re 1 V µPa^−1^) connected to a Tascam recorder (TASCAM DR-05X, Milton Keynes, UK). The fish were hand-held in seawater at a depth of 15 cm and at a distance of 5 cm from the hydrophone (the fish’s mouth was oriented towards the hydrophone) with the spinous dorsal fin blocked. All tested fish produced sound using this methodology; about 60 sounds were recorded for each fish. From these sounds, the 20 best-quality sounds (i.e., with the highest signal-to-noise ratios) were selected for the analyses. In very few cases where specimens were not cooperative, fewer sounds were recorded. In total, 7662 sounds were recorded and analyzed for the 388 individuals (mean of 19.4 sounds per specimen) (Supplementary Table 27).

### Sound analysis

Sounds were manually investigated using the software Avisoft-SAS Lab Pro 5.2.13 (Avisoft Bioacoustics, Glienicke, Germany). The sounds were digitalized at 44.1 kHz (16-bit resolution) and then band-pass filtered (50–4000 Hz). Then, six standard acoustic variables were measured from sounds (Fig. 2a, b): (1) sound duration (milliseconds, ms), (2) number of pulses in sounds, (3) pulse periods (measured as the peak-to-peak intervals between two consecutive pulses, ms), 4) duration of the last pulse (ms) based on oscillograms; we chose to measure this pulse in particular because its duration is the most reliable since it cannot be masked by any subsequent pulse in the sounds, (5) fundamental frequency (defined as the primary frequency of a harmonic sound, Hz) and (6) dominant frequency (defined as the frequency with the highest energy, Hz) that were extracted from the power spectrum of the whole sound (Hamming window; zero padding adapted to set the resolution to ~5 Hz). In some sounds, the pulses were not emitted all at once (Fig. 2a) but appeared to form a pattern where the pulses are grouped by blocks (sounds with pattern; Fig. 2b). In this case, three additional variables were extracted: (7) the interval (defined as the peak-to-peak duration between the last pulse of a block and the first pulse of the next block, ms), (8) the number of blocks composing the sound, 9) the number of pulses composing each block. Intervals were extracted from period measurements; a period was considered an interval in sounds when it was greater than 1.25 times the mean pulse period. We selected the value of 1.25 from several options (1.15, 1.20, 1.25, 1.30, 1.35, 1.40) because it best aligned the number of blocks calculated in a sample of one hundred randomly chosen sounds with the number of blocks visually observed from the same sounds. The last pulse period was not included as an interval even when it met this criterion, because it influenced mainly short sounds. In such cases, the number of periods is insufficient for the application of our system, which relies on calculating the average period. Therefore, sounds composed of ≤ 3 pulses consisted of one block and were automatically considered without a pattern. Finally, for each individual, we measured 10) the percentage of sounds with pattern produced among the ~20 recorded sounds. Sounds were then divided into 4 groups: (P1) sounds made of only 1 pulse, (P2) sounds made of 2 pulses, (P3) sounds composed of >2 pulses without pattern and (P4) sounds composed of >3 pulses with pattern. Depending on the group, some acoustical variables could not be measured.

### Statistical analysis

All analyses were performed in RStudio version 2023.9.0.463. Descriptive statistics were calculated for each temporal and spectral property of the acoustic signals produced by each individual. To investigate sound variation among the different taxonomic levels, subfamilies, genera, or species were used as grouping variables.

Since different acoustical features (sound duration, number of pulses in sounds, duration of the last pulse, dominant frequency) were known to be influenced by body size in holocentrids^39^, regressions were used to assess the relationships between TL and these acoustical parameters. The objective of this approach was to ascertain whether the various physical values characterizing sounds could be used directly or if they required adjustment for size.

To determine if sounds could be discriminated at different taxonomic levels based on acoustical features, univariate statistical analyses were first performed at the (1) subfamily level and at the (2) genus level (both including all genera and then specifically excluding the genus *Myripristis*). Then, differences in the acoustical variables of sounds of the (3) two main *Myripristis* branches in the existing phylogenetic tree (Fig. 1a) were investigated. Due to the high numbers of acoustical variables and species investigated, univariate analyses were, at the species level, only performed (4) within each genus and (5) within each of the two *Myripristis* branches.

For each taxonomic level investigated, the normality of the data and the homoscedasticity of the variances were first assessed to determine whether parametric or non-parametric tests should be used to perform the statistical analyses, respectively using Shapiro–Wilk tests and Bartlett’s tests, with significance level *p* < 0.05. Prior to these analyses, data were log- or square root-transformed if their transformation allowed to meet both criteria (normality and homoscedasticity). For all analyses, the tests used (*t* tests, Wilcoxon–Mann–Whitney tests, ANOVA, Kruskal–Wallis tests, post-hoc Tukey’s tests with a significance level of *p* < 0.05; and post-hoc Dunn’s tests with Benjamini–Hochberg correction with a significance level of 0.025 [*α*/2, since we used the parameter altp = FALSE in the dunn.test function]) were chosen accordingly.

PCAs were performed for each of the five taxonomic levels, similarly to univariate statistical analyses, except for *Holocentrus* because this genus is composed of only two species whose calls were already largely differentiated by the univariate statistics. For the interpretation of PCA results, we considered the number of factors equivalent to the number of eigenvalues greater than 1.0^8,68^. Convex hulls were built for each group in the different scatterplots to assess the capacity of taxa to optimally utilize acoustic traits, thereby showing their ability to exploit acoustic space. 3D CHs were represented in the 3D scatterplots using the cxhull function of the cxhull package. Their volumes were calculated for the subfamily, genus, and *Myripristis* group levels, using the convhulln function of the geometry package. Relative CH volumes were computed through two distinct methodologies: (1) first, by dividing the absolute CH volumes by the number of species within each tested taxon and (2) by dividing the absolute CH volumes by the number of individuals within each tested taxon. The first approach aimed to ascertain if the CH size of a taxon correlates with its species richness, under the hypothesis that taxa with a greater number of species might exploit the acoustic space more effectively. The second approach aimed to account for disparities in sample sizes across taxa. This adjustment was necessary since a larger number of specimens recorded within a particular taxon could have increased the variability for this taxon, thereby influencing the size of the CH. 3D scatterplots created using the first three PCs from the PCAs can be found in Supplementary Videos 1–4.

Finally, FDAs were conducted to test whether taxa (subfamilies, genera, etc.) can be correctly classified according to the acoustic traits. This analysis can be performed without assuming data normality^69^. The FDA calculates misclassification error rates and subsequently determines CCRs. These rates indicate the likelihood of making erroneous or accurate predictions about the classification of a given category based on the variables, for each taxonomic level under investigation. Confusion matrices were then built from these analyses for (1) all species, (2) *Myripristis* species, (3) species within each *Myripristis* group, and (4) species within each of the Holocentrinae genera. The FDAs were performed using the same variables as those used in the PCAs.

For both univariate and multivariate analyses, depending on the taxonomic level under investigation, from 6 to 10 acoustical variables were taken into consideration to perform the analyses since (a) some values were non-existent in the dataset (e.g., interval and number of pulses in blocks when an individual only produces sounds without pattern; values for interval are only available for 182 individuals out of 388) and (b) the 4 variables related to pattern (percentage of sounds with pattern, number of blocks in sounds, number of pulses in blocks and interval) were mainly related to the Myripristinae. Therefore, 8 acoustical variables (all except the interval and the number of pulses in blocks) were used to compare sounds of the two subfamilies, the 5 genera, *Myripristis* species of both groups and the 33 species, while the 6 variables unrelated to the pattern were used to investigate differences between species within each Holocentrinae genus and within *Myripristis* of group 1 (first branch of the tree). All 10 variables were only used to investigate species among *Myripristis* of group 2 (second branch of the tree).

## Supplementary information

Supplementary Information

Supplementary Video 1

Supplementary Video 2

Supplementary Video 3

Supplementary Video 4

## Data Availability

The dataset generated and analyzed during the current study is available in the “https://figshare.com/ repository, 10.6084/m9.figshare.25441594.
