# Supplementary material for: Sounds as taxonomic indicators in Holocentrid fishes
Source: NPJ Biodivers. 2024 Nov 5;3:33. doi: 10.1038/s44185-024-00064-4 (PMC11538288; doi:10.1038/s44185-024-00064-4)
Supplement: Supplementary file 1 — Supplementary Information [file 44185_2024_64_MOESM1_ESM.pdf]

Supplementary Notes 1.| Descriptions of the sounds produced by each species.

Species are presented individually and are organized by genus. Since we previously observed that sounds with pattern (sounds made of several blocks of pulses) (Fig. 3b) were mainly produced by *Myripristis* species of group 2 (Fig. 1a,b), variables related to pattern were only described for this group. An oscillogram of a representative sound is also available for each species in Supplementary Fig. 1.

***Myripristis* genus**

Using acoustical features of sounds, two distinct groups of *Myripristis* were formed with species of group 1 producing sounds that rarely exhibited a pattern (Fig. 2a) and species of group 2 producing many sounds that did show a pattern (Fig. 2b). These two groups corresponded to the two *Myripristis* branches in the holocentrid phylogenetic tree (Fig. 1a). *Myripristis pralinia, M. vittata, M. kuntee* and *M. jacobus* belonged to the first group, while *M. berndti, M. amaena, M. hexagona, M. adusta, M. murdjan, M. seychellensis* and *M. violacea* belonged to the second group. Sounds of species from group 1 will first be described, followed by the descriptions of the sounds produced by species of group 2. The position of *M. seychellensis* in the phylogenetic tree is unknown. Since its sounds were often made of pattern, as was the case for all species of the second group 2, it was considered as part of this group.

Group 1

<u>Myripristis jacobus</u>

Sounds of *M. jacobus* (N = 3, n = 60) are characterized by a duration of 46.7 ± 21.7 ms and are made of 3 to 4 pulses. The duration of the last pulse is comprised between 6 and 11 ms, and the period between 11 and 25 ms. Their fundamental frequency is quite low, 74 ± 26 Hz. The dominant frequency of sounds is 275 ± 38 Hz.

<u>Myripristis kuntee</u>

Sounds of *M. kuntee* (N = 25, n = 500) last between 40 and 80 ms and are composed of 4 to 7 pulses. Pulse period varies between 7 and 18 ms and the duration of the last pulse between 6 and 28 ms. Their fundamental frequency similarly varies quite importantly, between 67 and 139 Hz. Their dominant frequency is 265 ± 45 Hz.

<u>Myripristis pralinia</u>

The duration of *M. pralinia* sounds (N = 26, n = 520) varies between 51 and 100 ms and they are composed of 4 to 8 pulses, with a pulse period lasting from 9 to 15 ms. The duration of the last pulse greatly varies, from 7 to 27 ms. The fundamental frequency is between 72 and 121 Hz while the dominant frequency is 265 ± 42 Hz.

<u>Myripristis vittata</u>

Sounds of *M. vittata* (N = 7, n = 140) consist of 6 to 9 pulses, lasting between 77 and 118 ms. Duration of the last pulse varies between 11 and 24 ms. Pulse period is 11.6 ± 1.4 ms. The fundamental and dominant frequencies are 93 ± 10 Hz and 307 ± 38 Hz, respectively.

Group 2

<u>Myripristis adusta</u>

Myripristis adusta produces sounds composed of 4 to 10 pulses, lasting between 39 and 100 ms (N = 21, n = 420). This species produces a very high number of sounds with pattern (68 ± 31%). Those are composed of 2 to 3 blocks made of 2 to 4 pulses each, with an interval of 15.4 ± 4 ms. The pulse period lasts 6.9 ± 1.1 ms and the last pulse between 6 and 21 ms. The fundamental and dominant frequencies are 143 ± 25 Hz and 231 ± 51 Hz, respectively.

Myripristis amaena

Sounds produced by *M. amaena* (N = 17, n = 340) last between 60 and 145 ms and are made of 5 to 14 pulses, with a pulse period of 9.2 ± 1.4 ms. Half of the sounds are with pattern (48 ± 39%). These sounds are made of 2 to 4 blocks composed of 2 to 6 pulses each, with an interval of 15.7 ± 3.5 ms. The duration of the last pulse varies greatly, from 5 to 26 ms. The fundamental and dominant frequencies are 116 ± 17 Hz and 289 ± 32 Hz, respectively.

Myripristis berndti

Myripristis berndti produces sounds lasting between 58 and 112 ms and composed of 5 to 11 pulses (N = 21, n = 420). Forty-three percent (43 ± 37%) of them exhibit a pattern in which 2 to 3 blocks made of 2 to 4 pulses each are separated by an interval of 16.4 ± 2.3 ms. Pulse period is 9.3 ± 1.9 ms and the duration of the last pulse varies between 5 and 26 ms. They have fundamental and dominant frequencies similar to *M. amaena*, 115 ± 17 Hz and 287 ± 80 Hz, respectively.

Myripristis hexagona

Sounds produced by *M. hexagona* (N = 12, n = 240) consist of 5 to 11 pulses and last 42 to 94 ms with a pulse period of 6.8 ± 1.4 ms. A bit more than half of the sounds have pattern (52 ± 33%) and are made of 2 to 3 blocks composed of 2 to 3 pulses each, with an interval of 15.4 ± 5.1 ms. The duration of the last pulse ranges between 7 and 29 ms. The fundamental frequency is 145 ± 34 Hz and the dominant frequency is 241 ± 30 Hz.

Myripristis murdjan

Myripristis murdjan produces sounds composed of 4 to 10 pulses lasting 66 to 112 ms (N = 16, n = 320). Sixty-four percent (64 ± 29) of them are composed of 2 to 3 blocks made of 2 to 4 pulses each, with an interval of 16.3 ± 1.4 ms. The pulse period lasts 9.8 ± 3 ms and the duration

of last pulse ranges between 7 and 28 ms. The fundamental and dominant frequencies are 111 ± 19 Hz and 274 ± 56 Hz, respectively.

Myripristis violacea

Sounds of *M. violacea* last 49 to 103 ms and consist of 4 to 8 pulses (N = 22, n = 440). This species also produces a very high number of sounds with pattern (59 ± 30%), which are mainly made of 2 to 3 blocks composed of 2 to 4 pulses each and have an interval of 14.6 ± 3.2 ms. The pulse period is 8.4 ± 1.1 ms and the duration of the last pulse is comprised between 5 and 26 ms. The fundamental and dominant frequencies are 122 ± 13 Hz and 226 ± 66 Hz, respectively.

Myripristis seychellensis

Myripristis seychellensis produce sounds lasting between 50 and 78 ms and composed of 3 to 5 pulses (N = 6, n = 119). This species produced the highest number of sounds with pattern (83 ± 19%). Pulses of these sounds are mainly grouped in 2 to 3 blocks of 2 pulses each, with an interval of 23.8 ± 3 ms. The duration of the last pulse is between 12 and 25 ms and pulse period is 8.7 ± 3.6 ms. The fundamental and dominant frequencies are 136 ± 17 Hz and 209 ± 65 Hz, respectively.

***Sargocentron* genus**

Sargocentron caudimaculatum

Sounds of *S. caudimaculatum* (N = 23, n = 451) have a duration comprised between 40 and 82 ms and are composed of 2 to 5 pulses, whose last pulse ranges between 16 and 29 ms. Pulse period is 14.6 ± 1.7 ms. Their fundamental frequency is quite low (66 ± 6 Hz). The dominant frequency is 272 ± 50 Hz.

Sargocentron cornutum

Sounds of *S. cornutum* (N = 6, n = 120) are composed of 1 to 6 pulses, lasting between 44 and 62 ms, with a period of 8 ± 0.4 ms and a fundamental frequency of 124 ± 6 Hz. The dominant frequency is 286 ± 31 Hz. The duration of the last pulse in sounds varies between 10 and 22 ms.

Sargocentron dorsomaculatum

Sounds of *S. dorsomaculatum* (N = 2, n = 40) are always made up of 5 pulses and last 50.9 ± 1 ms. Their last pulse has a duration that ranges between 12 and 16 ms and their pulse period is 8.4 ± 0.6 ms. The fundamental and dominant frequencies are 123 ± 4 Hz and 426 ± 80 Hz, respectively.

Sargocentron melanospilos

The duration of *S. melanospilos* (N = 6, n = 120) sounds is comprised between 38 and 63 ms and sounds are generally composed of 2 to 5 pulses. Their pulse period is 12.7 ± 2.1 ms and the duration of the last pulse is 15.1 ± 1.6 ms. They have a quite low fundamental frequency of 76 ± 9 Hz and their dominant frequency is 326 ± 22 Hz.

Sargocentron praslin

Sounds of *S. praslin* (N = 7, n = 140) are characterized by a duration of 48 to 61 ms, 4 to 6 pulses and a pulse period of 9.7 ± 1.5 ms. The last pulse ranges between 14 and 21 ms. The fundamental frequency is also quite low (98 ± 17 Hz) and the dominant frequency is 291 ± 29 Hz.

Sargocentron rubrum

Sounds of *S. rubrum* (N = 6, n = 120) are composed of 3 to 4 pulses and last between 49 and 74 ms. The pulse period is 11.4 ± 2 ms, and the duration of the last pulse is comprised between

18 and 34 ms. The fundamental and dominant frequencies are close to that of *S. praslin* (91 ± 16 Hz and 274 ± 26 Hz, respectively).

Sargocentron seychellense

Sounds of *S. seychellense* (N = 7, n = 140) are the shortest among *Sargocentron* species. They are usually made of 2 pulses (1.9 ± 0.4) and have a duration between 38 and 53 ms. The last pulse duration is quite long, between 23 and 35 ms. The pulse period is 16.9 ± 5.3 ms. These sounds have a low fundamental frequency of 62 ± 17 Hz, and a dominant frequency of 282 ± 37 Hz.

Sargocentron spiniferum

Sounds of *S. spiniferum* (N = 25, n = 485) are composed of 1 to 4 pulses and last between 31 and 80 ms, with a pulse period of 16.7 ± 3 ms and the last pulse that also lasts quite long (25.4 ± 4.4 ms). The fundamental frequency is very low (57 ± 10 Hz) and dominant frequency is 244 ± 30 Hz.

Sargocentron tiere

Sounds of *S. tiere* (N = 11, n = 220) are composed of 1 to 3 pulses and last between 29 and 86 ms. Their pulse period of 32 ± 6.7 ms is the longest among all species. Their last pulse is also very long (27.6 ± 3.4 ms). They are also characterized by their very low fundamental frequency comprised between 24 and 41 Hz. Their dominant frequency is 256 ± 32 Hz.

Sargocentron tiereoides

Sounds of *S. tiereoides* (N = 8, n = 160) consist of 4 to 6 pulses, lasting between 47 and 75 ms, with a period of 11.3 ± 1 ms and a duration of the last pulse in sounds that varies between 11 and 27 ms. The fundamental and dominant frequencies are 93 ± 6 Hz and 384 ± 119 Hz, respectively.

Sargocentron violaceum

Sargocentron violaceum sounds (N = 7, n = 140) are characterized by a duration of 50 to 70 ms and possess 4 to 6 pulses, with a pulse period of 11 ± 0.9 ms and a quite short and steady final pulse (14.5 ± 0.8 ms). Their fundamental and dominant frequencies are similar to those of *S. tiereoides,* 95 ± 8 Hz and 339 ± 30 Hz, respectively.

***Flammeo* genus**

Flammeo marianus

Flammeo marianus sounds (N = 4, n = 76) are characterized by a duration of 18.1 ± 2.2 ms and are mainly composed of a single pulse (1.2 ± 0.1). In rare cases where the sounds are made of more pulses, they invariably form a single block of pulses and therefore do not exhibit any pattern. In these pulsed sounds, the pulse period ranges between 9 and 11 ms. Their fundamental and dominant frequencies are 100 ± 4 Hz and 358 ± 47 Hz, respectively. The last pulse of the sound lasts between 11.1 and 14.2 ms.

***Holocentrus* genus**

Similarly to sounds of *F. marianus, Holocentrus* species sounds are always made of one block of pulses and therefore do not display pattern.

Holocentrus adscensionis

Holocentrus adscensionis sounds (N = 5, n = 100) are composed of 3 to 5 pulses and last between 41 and 62 ms. Their fundamental and dominant frequencies are 94 ± 19 Hz and 344 ± 78 Hz, respectively, and their pulse period varies between 9 and 13 ms. The duration of their last pulse is comprised between 9 and 11 ms.

Holocentrus rufus

Holocentrus rufus sounds (N = 4, n = 80) are composed of 4 to 5 pulses and last between 28 and 41 ms. The fundamental and dominant frequencies are 142 ± 12 Hz and 551 ± 57 Hz,

respectively, and the pulse period lasts between 7 and 8 ms. The final pulse of the sounds lasts between 5 and 8 ms.

***Neoniphon* genus**

Neoniphon argenteus

Sounds of *N. argenteus* (N = 10, n = 200) consist of 4 to 8 pulses, lasting from 40 to 80 ms, with a pulse period of 8.3 ± 1 ms and a fundamental frequency of 125 ± 13 Hz. Dominant frequency varies between 329 and 778 Hz, and the duration of the last pulse in the sounds is comprised between 4 and 22 ms.

Neoniphon coruscum

Sounds of *N. coruscum* (N = 4, n = 80) are among the shortest. They are generally composed of 1 to 3 pulses and last between 14 and 37 ms, with a period of 13.2 ± 1.1 ms and a low fundamental frequency of 79 ± 15 Hz. The duration of the last pulse is also short and steady across sounds (10.8 ± 0.9 ms). The dominant frequency is 490 ± 54 Hz.

Neoniphon diadema

Sounds of *N. diadema* (N = 25, n = 480) last 12 to 46 ms and are composed of 1 to 4 pulses. The duration of their last pulse greatly varies, from 9 to 34 ms. The pulse period is 10 ± 2 ms and the fundamental frequency is 99 ± 18 Hz, while the dominant frequency is 399 ± 59 Hz.

Neoniphon microstoma

Neoniphon microstoma sounds (N = 11, n = 180) are those, among all species, that vary the most in duration. They last between 29 and 101 ms and consist of 3 to 8 pulses, with a pulse period of 10.6 ± 0.7 ms. The fundamental frequency is 97 ± 7 Hz and the the dominant frequency is 379 ± 90 Hz. The final pulse in sounds lasts between 9 and 22 ms.

Neoniphon opercularis

Sounds of *N. opercularis* (N = 9, n = 180) consist of 4 to 9 pulses, lasting 58 to 87 ms with a pulse period of 9.9 ± 2.1 ms. The final pulse varies in duration between 10 and 25 ms. The fundamental and dominant frequencies are is 109 ± 23 Hz and 364 ± 71 Hz, respectively.

Neoniphon punctatissimum

Sounds of *N. punctatissimum* (N = 5, n = 91) consist of very short sounds, mainly made of a single pulse and lasting between 9 and 28 ms. Among the 91 sounds that were analyzed in this study, only one was made of 2 pulses and its pulse period and last pulse duration were 22 ms and 13 ms, respectively. The dominant frequency varies between 272 and 539 Hz.

Neoniphon sammara

Sounds of *N. sammara* (N = 25, n = 500) are characterized by a duration of 37 to 78 ms and 4 to 8 pulses, with a pulse period of 7.5 ± 0.6 ms. The final pulse lasts between 5 and 21 ms. The fundamental and dominant frequencies are 132 ± 9 Hz and 382 ± 134 Hz, respectively.

Neoniphon vexillarium

Sounds of *N. vexillarium* (N = 2, n = 40) are the shortest, lasting between 12 and 15 ms, and always composed of a single pulse. The dominant frequency is 420 ± 145 Hz.

Supplementary Table 1. Summary of the number of sounds made of 1 (P1), 2 (P2) and >2 pulses without pattern (P3) and >3 pulses with pattern (P4) for each species. n = number of sounds, N = number of individuals, nSP = number of species.

Species	Number of sounds P1	Number of sounds P2	Number of sounds P3	Number of sounds P4
F. marianus	63	12	1	
H. adscensionis			100	
H. rufus			80	
M. adusta		3	132	285
M. amaena			178	162
M. berndti		1	240	179
M. hexagona		1	113	126
M. jacobus	1	9	50	
M. kuntee		1	397	102
M. murdjan	1	2	111	206
M. pralinia			512	8
M. seychellensis		14	6	99
M. violacea			181	259
M. vittata			139	1
N. argenteus		2	184	14
N. coruscum	72		1	7
N. diadema	318	120	31	11
N. microstoma	10	50	80	40
N. opercularis			178	2
N. punctatissimum	89	1	1	
N. sammara		6	449	45
N. vexillarium	40			
S. caudimaculatum	3	36	408	4
S. cornutum	40		76	4
S. dorsomaculatum			36	4
S. melanospilos		32	71	17
S. praslin		2	115	23
S. rubrum		17	97	6
S. seychellense	40	91	9	
S. spiniferum	38	121	323	3
S. tiere	64	118	38	
S. tiereoides			159	1
S. violaceum			139	1
**n (N)**	**779 (75)**	**639 (104)**	**4635 (334)**	**1609 (186)**
**nSP**	**13**	**20**	**32**	**25**

Supplementary Table 2. Summary of mean ± sd and [min – max values], calculated from individual means, for the different acoustical variables for each subfamily. N = number of specimens recorded; n = number of sounds recorded for all individuals. F0 = fundamental frequency. *For sounds with pattern.

Subfamily	Sound duration (ms)	Number of pulses	F0 (Hz)	Dominant frequency (Hz)	Duration of the last pulse (ms)	Number of blocks in the sound	% Sounds with pattern	Number of pulses per block*	Period (ms)	Interval (ms)
**Holocentrinae** (N = 212, n = 4143)	50.4 ± 18 [8.7 - 100.9]	3.7 ± 1.9 [1 - 8.7]	90 ± 31 [24 - 158]	343 ± 108 [173 - 831]	18.7 ± 6.9 [4.2 - 35.4]	1 ± 0.1 [1 - 1.6]	4 ± 9 [0 - 45]	2.9 ± 0.9 [1.5 - 6.5]	12.7 ± 6.1 [6.3 - 45]	15.1 ± 4.7 [8.2 - 26.7]
**Myripristinae** (N = 176, n = 3519)	73.9 ± 17.9 [32 - 145.2]	6.4 ± 1.7 [3 - 13.6]	115 ± 25 [47 - 220]	260 ± 58 [106 - 544]	17.3 ± 6.4 [4.5 - 28.9]	1.6 ± 0.7 [1 - 4]	41 ± 37 [0 - 100]	2.7 ± 0.6 [1.6 - 5.7]	9.5 ± 2.7 [4.2 - 24.9]	16.7 ± 4 [9.1 - 29.5]

Supplementary Table 3. Summary of mean ± sd and [min – max values], calculated from individual means, for the different acoustical variables for each genus. N = number of specimens recorded; n = number of sounds recorded for all individuals. F0 = fundamental frequency. *For sounds with pattern.

Genus	Sound duration (ms)	Number of pulses	F0 (Hz)	Dominant frequency (Hz)	Duration of the last pulse (ms)	Number of blocks in the sound	% Sounds with pattern	Number of pulses per block*	Period (ms)	Interval (ms)
Flammeo (N = 4, n = 76)	18.1 ± 2.2 [15.5 - 21]	1.2 ± 0.1 [1 - 1.3]	100 ± 4 [97 - 102]	358 ± 47 [313 - 407]	12.8 ± 1.6 [11.1 - 14.2]	1 ± 0 [1 - 1]	0 ± 0 [0 - 0]		10.1 ± 1.1 [8.9 - 11]	
Holocentrus (N = 9, n = 180)	41.3 ± 11 [27.8 - 61.6]	4.3 ± 0.6 [3.3 - 5]	115 ± 30 [77 - 158]	436 ± 127 [259 - 610]	8.6 ± 2.4 [4.9 - 11.4]	1 ± 0 [1 - 1]	0 ± 0 [0 - 0]		9.7 ± 2.6 [6.5 - 13.3]	
Myripristis (N = 176, n = 3519)	73.9 ± 17.9 [32 - 145.2]	6.4 ± 1.7 [3 - 13.6]	115 ± 25 [47 - 220]	260 ± 58 [106 - 544]	17.3 ± 6.4 [4.5 - 28.9]	1.6 ± 0.7 [1 - 4]	41 ± 37 [0 - 100]	2.7 ± 0.6 [1.6 - 5.7]	9.5 ± 2.7 [4.2 - 24.9]	16.7 ± 4 [9.1 - 29.5]
Neoniphon (N = 91, n = 1751)	45.1 ± 21.6 [8.7 - 100.9]	4.1 ± 2.5 [1 - 8.7]	113 ± 21 [60 - 158]	402 ± 113 [233 - 831]	15.6 ± 5.8 [4.2 - 33.8]	1.1 ± 0.1 [1 - 1.5]	7 ± 11 [0 - 45]	3.2 ± 1 [1.5 - 6.5]	9.4 ± 2.4 [6.3 - 22]	15.1 ± 4.7 [8.3 - 26.7]
Sargocentron (N = 108, n = 2136)	56.8 ± 11.2 [28.5 - 86.1]	3.5 ± 1.3 [1 - 6]	72 ± 25 [24 - 130]	285 ± 63 [173 - 529]	22 ± 5.9 [10.3 - 35.4]	1 ± 0.1 [1 - 1.6]	3 ± 7 [0 - 40]	2.5 ± 0.5 [2 - 4.5]	15.6 ± 6.9 [7.7 - 45]	15 ± 4.7 [8.2 - 24.8]

Supplementary Table 4. Summary of mean ± sd and [min – max values], calculated from individual means, for the different acoustical variables for each *Myripristis* group. N = number of specimens recorded; n = number of sounds recorded for all individuals. F0 = fundamental frequency. *For sounds with pattern.

Myripristis group	Sound duration (ms)	Number of pulses	F0 (Hz)	Dominant frequency (Hz)	Duration of the last pulse (ms)	Number of blocks in the sound	% Sounds with pattern	Number of pulses per block*	Period (ms)	Interval (ms)
1 (N = 61, n = 1220)	70.9 ± 15.8 [32 - 117.9]	5.6 ± 1.3 [3 - 9.2]	97 ± 15 [47 - 139]	270 ± 44 [197 - 390]	17.6 ± 6.3 [5.5 - 27.9]	1.1 ± 0.3 [1 - 2.6]	9 ± 19 [0 - 90]	2.3 ± 0.3 [1.6 - 2.8]	11.5 ± 2.6 [7.1 - 24.9]	19.9 ± 3.7 [14.3 - 29.5]
2 (N = 115, n = 2299)	75.5 ± 18.7 [39.1 - 145.2]	6.8 ± 1.7 [3 - 13.6]	125 ± 24 [64 - 220]	255 ± 63 [106 - 544]	17.1 ± 6.5 [4.5 - 28.9]	1.9 ± 0.7 [1 - 4]	57 ± 34 [0 - 100]	2.7 ± 0.7 [1.7 - 5.7]	8.5 ± 2.1 [4.2 - 19.6]	16.1 ± 3.8 [9.1 - 28.4]

Supplementary Table 5. Summary of mean ± sd and [min – max values], calculated from individual means, for the different acoustical variables for each species. N = number of specimens recorded; n = number of sounds recorded for all individuals. F0 = fundamental frequency. *For sounds with pattern.

Species	Sound duration (ms)	Number of pulses	F0 (Hz)	Dominant frequency (Hz)	Duration of the last pulse (ms)	Number of blocks in the sound	% Sounds with pattern	Number of pulses per block*	Period (ms)	Interval (ms)
F. marianus<br>(N = 4, n = 76)	18.1 ± 2.2<br>[15.5 - 21]	1.2 ± 0.1<br>[1 - 1.3]	100 ± 4<br>[97 - 102]	358 ± 47<br>[313 - 407]	12.8 ± 1.6<br>[11.1 - 14.2]	1 ± 0<br>[1 - 1]	0 ± 0<br>[0 - 0]		10.1 ± 1.1<br>[8.9 - 11]	
H. adscensionis<br>(N = 5, n = 100)	48.1 ± 9.2<br>[40.7 - 61.6]	4.3 ± 0.7<br>[3.3 - 5]	94 ± 19<br>[77 - 125]	344 ± 78<br>[259 - 448]	10.3 ± 0.9<br>[9 - 11.4]	1 ± 0<br>[1 - 1]	0 ± 0<br>[0 - 0]		11.6 ± 1.9<br>[8.6 - 13.3]	
H. rufus<br>(N = 4, n = 80)	32.8 ± 6.1<br>[27.8 - 41.3]	4.3 ± 0.5<br>[4 - 5]	142 ± 12<br>[131 - 158]	551 ± 57<br>[500 - 610]	6.4 ± 1.4<br>[4.9 - 7.9]	1 ± 0<br>[1 - 1]	0 ± 0<br>[0 - 0]		7.4 ± 0.7<br>[6.5 - 7.9]	
M. adusta<br>(N = 21, n = 420)	65.7 ± 15.9<br>[39.1 - 96.7]	6.5 ± 1.4<br>[4.3 - 9.8]	143 ± 25<br>[106 - 196]	231 ± 51<br>[142 - 333]	14.9 ± 4.3<br>[5.8 - 21.2]	2.1 ± 0.6<br>[1 - 3.1]	68 ± 31<br>[0 - 100]	2.6 ± 0.4<br>[2 - 3.5]	6.9 ± 1.1<br>[5 - 8.9]	15.4 ± 4<br>[9.4 - 24.2]
M. amaena<br>(N = 17, n = 340)	91.8 ± 20.6<br>[60 - 145.2]	8 ± 2.1<br>[5.2 - 13.6]	116 ± 17<br>[83 - 146]	289 ± 32<br>[240 - 362]	18.4 ± 6.5<br>[4.7 - 25.7]	1.9 ± 0.9<br>[1 - 4]	48 ± 39<br>[0 - 100]	3.2 ± 1.1<br>[2 - 5.7]	9.2 ± 1.4<br>[6.7 - 12.8]	15.7 ± 3.5<br>[11.6 - 25.5]
M. berndti<br>(N = 21, n = 420)	80.5 ± 14.7<br>[57.4 - 111.9]	7.2 ± 1.6<br>[5.1 - 10.6]	115 ± 17<br>[82 - 138]	287 ± 80<br>[184 - 544]	17.9 ± 6<br>[4.7 - 26]	1.6 ± 0.6<br>[1 - 2.6]	43 ± 37<br>[0 - 90]	3 ± 0.7<br>[2 - 4.1]	9.3 ± 1.9<br>[7.3 - 14.7]	16.4 ± 2.3<br>[11.6 - 19.4]
M. hexagona<br>(N = 12, n = 240)	65.6 ± 15.4<br>[41.9 - 93.6]	6.3 ± 0.9<br>[5.2 - 8.6]	145 ± 34<br>[118 - 220]	241 ± 30<br>[166 - 280]	20.1 ± 7.4<br>[7 - 28.9]	1.7 ± 0.5<br>[1 - 2.5]	52 ± 33<br>[0 - 95]	2.6 ± 0.3<br>[2.2 - 3.2]	6.8 ± 1.4<br>[4.2 - 8]	15.4 ± 5.1<br>[10.2 - 28.4]
M. jacobus<br>(N = 3, n = 60)	46.7 ± 21.7<br>[32 - 71.6]	3.3 ± 0.3<br>[3 - 3.6]	74 ± 26<br>[47 - 99]	275 ± 38<br>[233 - 308]	9 ± 2.3<br>[6.4 - 10.6]	1 ± 0<br>[1 - 1]	0 ± 0<br>[0 - 0]		16.3 ± 7.6<br>[10.4 - 24.9]	
M. kuntee<br>(N = 25, n = 500)	64.1 ± 12.4<br>[40.1 - 84.1]	4.9 ± 0.9<br>[3.7 - 7.4]	96 ± 17<br>[67 - 139]	265 ± 45<br>[197 - 390]	17.3 ± 6.7<br>[5.5 - 27.9]	1.2 ± 0.4<br>[1 - 2.6]	20 ± 25<br>[0 - 90]	2.2 ± 0.3<br>[1.6 - 2.8]	11.7 ± 2.4<br>[7.1 - 17.8]	19.8 ± 4<br>[14.3 - 29.5]
M. murdjan<br>(N = 16, n = 320)	88.3 ± 13.5<br>[65.5 - 112.1]	7.4 ± 1.9<br>[3.5 - 10.3]	111 ± 19<br>[64 - 136]	274 ± 56<br>[194 - 365]	18.4 ± 6.8<br>[6.6 - 27.5]	2.1 ± 0.7<br>[1 - 3.4]	64 ± 29<br>[0 - 100]	2.9 ± 0.5<br>[1.9 - 4.1]	9.8 ± 3<br>[7.3 - 19.6]	16.3 ± 1.4<br>[14.4 - 18.4]
M. pralinia<br>(N = 26, n = 520)	74.6 ± 10.4<br>[50.7 - 100.1]	6.1 ± 0.8<br>[4 - 7.5]	101 ± 12<br>[72 - 121]	265 ± 42<br>[200 - 379]	18.7 ± 6<br>[6.7 - 27.1]	1 ± 0.1<br>[1 - 1.4]	2 ± 7<br>[0 - 35]	2.6 ± 0.1<br>[2.5 - 2.7]	10.8 ± 1.5<br>[8.9 - 15.3]	21.6 ± 1.4<br>[20.6 - 22.6]

Species	Sound duration (ms)	Number of pulses	F0 (Hz)	Dominant frequency (Hz)	Duration of the last pulse (ms)	Number of blocks in the sound	% Sounds with pattern	Number of pulses per block*	Period (ms)	Interval (ms)
M. seychellensis<br>(N = 6, n = 119)	63.5 ± 11.7<br>[49.7 - 77.8]	4.3 ± 1<br>[3 - 5.3]	136 ± 17<br>[105 - 148]	209 ± 65<br>[142 - 288]	17.1 ± 5.3<br>[12 - 25.4]	2.1 ± 0.5<br>[1.6 - 2.7]	83 ± 19<br>[55 - 100]	1.9 ± 0.1<br>[1.7 - 2]	8.7 ± 3.6<br>[6.8 - 16]	23.8 ± 3<br>[18.7 - 27.8]
M. violacea<br>(N = 22, n = 440)	66.8 ± 14.4<br>[48.7 - 103]	6.3 ± 1.2<br>[3.9 - 8.2]	122 ± 13<br>[98 - 161]	226 ± 66<br>[106 - 342]	14.7 ± 7.4<br>[4.5 - 26.2]	1.9 ± 0.6<br>[1 - 3]	59 ± 30<br>[0 - 100]	2.6 ± 0.5<br>[1.7 - 3.7]	8.4 ± 1.1<br>[6.8 - 10.6]	14.6 ± 3.2<br>[9.1 - 20.8]
M. vittata<br>(N = 7, n = 140)	91.8 ± 15.1<br>[77 - 117.9]	7.1 ± 1.1<br>[5.8 - 9.2]	93 ± 10<br>[83 - 107]	307 ± 38<br>[260 - 365]	18.3 ± 4.3<br>[11.4 - 23.5]	1 ± 0<br>[1 - 1]	1 ± 2<br>[0 - 5]	2.5<br>[2.5 - 2.5]	11.6 ± 1.4<br>[9.7 - 13.4]	18.1<br>[18.1 - 18.1]
N. argenteus<br>(N = 100, n = 200)	55.6 ± 11.5<br>[40 - 79.8]	5.9 ± 1.1<br>[4.4 - 7.6]	125 ± 13<br>[106 - 143]	496 ± 175<br>[329 - 778]	13.3 ± 7.2<br>[4.2 - 21.6]	1.1 ± 0.1<br>[1 - 1.2]	7 ± 9<br>[0 - 20]	4 ± 1.6<br>[2.7 - 6.5]	8.3 ± 1<br>[6.8 - 9.8]	12.8 ± 2.5<br>[9.3 - 15.5]
N. coruscum<br>(N = 4, n = 80)	21.7 ± 10.6<br>[13.5 - 37.2]	1.6 ± 0.8<br>[1 - 2.8]	79 ± 15<br>[62 - 88]	490 ± 54<br>[415 - 539]	10.8 ± 0.9<br>[10 - 11.7]	1 ± 0.1<br>[1 - 1.2]	9 ± 11<br>[0 - 25]	3.2 ± 0.6<br>[2.5 - 3.6]	13.2 ± 1.1<br>[12.5 - 14.4]	23.9 ± 3.4<br>[20.1 - 26.7]
N. diadema<br>(N = 25, n = 480)	27.2 ± 8.7<br>[11.8 - 46.3]	1.6 ± 0.8<br>[1 - 4.4]	99 ± 18<br>[60 - 127]	399 ± 59<br>[313 - 556]	18.7 ± 6.6<br>[8.7 - 33.8]	1 ± 0.1<br>[1 - 1.3]	3 ± 6<br>[0 - 20]	2.3 ± 0.8<br>[1.5 - 3.4]	10 ± 2<br>[6.4 - 14.1]	15.7 ± 4.7<br>[8.3 - 22.5]
N. microstoma<br>(N = 11, n = 180)	61.8 ± 24.3<br>[28.5 - 100.9]	5.3 ± 2.1<br>[2.5 - 8.1]	97 ± 7<br>[89 - 112]	379 ± 90<br>[233 - 569]	13.5 ± 4.3<br>[8.6 - 21.8]	1.3 ± 0.2<br>[1 - 1.5]	20 ± 16<br>[0 - 40]	3.3 ± 0.8<br>[2.3 - 4.8]	10.6 ± 0.7<br>[9.2 - 11.7]	18.6 ± 2.3<br>[14.2 - 21.1]
N. opercularis<br>(N = 9, n = 180)	76.4 ± 9.7<br>[57.9 - 87.4]	7.1 ± 1.4<br>[4.1 - 8.7]	109 ± 23<br>[64 - 132]	364 ± 71<br>[292 - 465]	16.2 ± 4.6<br>[10 - 24.5]	1 ± 0<br>[1 - 1.1]	1 ± 3<br>[0 - 10]	3.4<br>[3.4 - 3.4]	9.9 ± 2.1<br>[7.8 - 13.9]	10.8<br>[10.8 - 10.8]
N. punctatissimum<br>(N = 5, n = 91)	21.9 ± 7.6<br>[8.7 - 27.6]	1.1 ± 0.1<br>[1 - 1.3]		364 ± 107<br>[272 - 539]	13.2<br>[13.2 - 13.2]	1 ± 0<br>[1 - 1]	0 ± 0<br>[0 - 0]		22<br>[22 - 22]	
N. sammara<br>(N = 25, n = 500)	51 ± 11.4<br>[36.8 - 77.8]	5.4 ± 1.2<br>[3.6 - 7.5]	132 ± 9<br>[118 - 158]	382 ± 134<br>[250 - 831]	15.2 ± 4.6<br>[5 - 21.1]	1.1 ± 0.1<br>[1 - 1.4]	9 ± 12<br>[0 - 45]	3.2 ± 0.9<br>[1.9 - 5]	7.5 ± 0.6<br>[6.3 - 8.3]	12.1 ± 2.7<br>[9.4 - 19.4]
N. vexillarium<br>(N = 2, n = 40)	13.3 ± 2.5<br>[11.6 - 15.1]	1 ± 0<br>[1 - 1]		420 ± 145<br>[318 - 523]		1 ± 0<br>[1 - 1]	0 ± 0<br>[0 - 0]			
S. caudimaculatum<br>(N = 23, n = 451)	62.8 ± 11.4<br>[40.1 - 81.9]	3.8 ± 0.7<br>[2 - 5]	66 ± 6<br>[56 - 76]	272 ± 50<br>[173 - 362]	20.6 ± 3<br>[16.3 - 29.4]	1 ± 0<br>[1 - 1.1]	1 ± 2<br>[0 - 10]	2.3 ± 0.3<br>[2 - 2.5]	14.6 ± 1.7<br>[10.9 - 17.8]	17.2 ± 1.3<br>[15.7 - 18.2]
S. cornutum<br>(N = 6, n = 120)	51.7 ± 7.3<br>[43.7 - 61.7]	3.8 ± 2.2<br>[1 - 5.7]	124 ± 6<br>[116 - 130]	286 ± 31<br>[253 - 344]	18.1 ± 5.3<br>[10.3 - 22.2]	1 ± 0<br>[1 - 1.1]	3 ± 6<br>[0 - 15]	3.8 ± 1.1<br>[3 - 4.5]	8 ± 0.4<br>[7.7 - 8.5]	10.6 ± 1.8<br>[9.3 - 11.9]
S. dorsomaculatum<br>(N = 2, n = 40)	50.9 ± 1<br>[50.2 - 51.6]	5 ± 0<br>[5 - 5]	123 ± 4<br>[120 - 126]	426 ± 80<br>[369 - 482]	13.8 ± 2.8<br>[11.8 - 15.8]	1.1 ± 0.1<br>[1 - 1.2]	10 ± 7<br>[5 - 15]	2.4 ± 0.1<br>[2.3 - 2.5]	8.4 ± 0.6<br>[8 - 8.9]	11.9 ± 0.4<br>[11.7 - 12.2]

Species	Sound duration (ms)	Number of pulses	F0 (Hz)	Dominant frequency (Hz)	Duration of the last pulse (ms)	Number of blocks in the sound	% Sounds with pattern	Number of pulses per block*	Period (ms)	Interval (ms)
S. melanospilos<br>(N = 6, n = 120)	51.1 ± 11<br>[38.3 - 63.4]	3.8 ± 1.2<br>[2.4 - 5.2]	76 ± 9<br>[68 - 92]	326 ± 22<br>[307 - 366]	15.1 ± 1.6<br>[13.1 - 18]	1.2 ± 0.2<br>[1 - 1.4]	14 ± 11<br>[0 - 30]	2.3 ± 0.3<br>[2 - 2.8]	12.7 ± 2.1<br>[10.8 - 16.1]	18.6 ± 5.6<br>[12.6 - 24.8]
S. praslin<br>(N = 7, n = 140)	56.3 ± 4.3<br>[48.4 - 60.9]	4.7 ± 0.9<br>[3.7 - 5.8]	98 ± 17<br>[73 - 119]	291 ± 29<br>[259 - 347]	18.2 ± 2.9<br>[14.1 - 20.9]	1.2 ± 0.2<br>[1 - 1.6]	16 ± 13<br>[0 - 40]	2.5 ± 0.3<br>[2.3 - 3]	9.7 ± 1.5<br>[7.9 - 11.6]	11.8 ± 3<br>[8.2 - 16.1]
S. rubrum<br>(N = 6, n = 120)	59 ± 8.7<br>[48.5 - 74.1]	3.8 ± 0.5<br>[3 - 4.2]	91 ± 16<br>[72 - 111]	274 ± 26<br>[234 - 311]	26.4 ± 5.1<br>[18 - 33.9]	1 ± 0.1<br>[1 - 1.1]	5 ± 4<br>[0 - 10]	2.2 ± 0.2<br>[2 - 2.5]	11.4 ± 2<br>[8.9 - 13.5]	15.8 ± 6.4<br>[10 - 24.7]
S. seychellense<br>(N = 7, n = 140)	44.4 ± 5.9<br>[37.8 - 53.3]	1.9 ± 0.4<br>[1.3 - 2.6]	62 ± 17<br>[36 - 77]	282 ± 37<br>[247 - 347]	29.2 ± 5<br>[23.3 - 35.4]	1 ± 0<br>[1 - 1]	0 ± 0<br>[0 - 0]		16.9 ± 5.3<br>[12.5 - 25.5]	
S. spiniferum<br>(N = 25, n = 485)	55.1 ± 10.1<br>[31 - 79.6]	2.7 ± 0.7<br>[1.4 - 4.1]	57 ± 10<br>[40 - 75]	244 ± 30<br>[189 - 287]	25.4 ± 4.4<br>[15.9 - 33.8]	1 ± 0<br>[1 - 1.1]	1 ± 2<br>[0 - 10]	2.1 ± 0.1<br>[2 - 2.2]	16.7 ± 3<br>[12.6 - 23.5]	18.3 ± 4<br>[15.5 - 21.1]
S. tiere<br>(N = 11, n = 220)	55.3 ± 17.1<br>[28.5 - 86.1]	1.9 ± 0.5<br>[1 - 2.9]	33 ± 5<br>[24 - 41]	256 ± 32<br>[198 - 313]	27.6 ± 3.4<br>[24.6 - 34.5]	1 ± 0<br>[1 - 1]	0 ± 0<br>[0 - 0]		32 ± 6.7<br>[24 - 45]	
S. tiereoides<br>(N = 8, n = 160)	61 ± 9<br>[46.6 - 74.8]	4.7 ± 0.6<br>[4 - 5.8]	93 ± 6<br>[82 - 100]	384 ± 119<br>[242 - 529]	17.4 ± 5.3<br>[10.5 - 27.3]	1 ± 0<br>[1 - 1]	1 ± 2<br>[0 - 5]	3<br>[3 - 3]	11.3 ± 1<br>[10.1 - 13.1]	12.5<br>[12.5 - 12.5]
S. violaceum<br>(N = 7, n = 140)	62.7 ± 7.6<br>[50.4 - 70.1]	5.2 ± 0.6<br>[4.1 - 6]	95 ± 8<br>[85 - 107]	339 ± 30<br>[296 - 379]	14.5 ± 0.8<br>[13.5 - 16]	1 ± 0<br>[1 - 1]	1 ± 2<br>[0 - 5]	2<br>[2 - 2]	11 ± 0.9<br>[9.4 - 12.4]	16.2<br>[16.2 - 16.2]

Supplementary Table 6. Results of the regression analyses between sound duration (Du) and size (TL) for each species. *P* values in bold are < 0.05.

Species	Formula	Slope estimate	Intercept estimate	Multiple R^2	***P***
F. marianus	Du ~ TL	0.81	5.53	0.15	0.61
H. adscensionis		4.82	-78.08	0.63	0.42
H. rufus		1.73	12.05	0.64	0.2
M. adusta		-0.43	71.42	0.02	0.53
M. amaena		-0.01	4.66	0.05	0.41
M. berndti		0.15	78.43	0	0.83
M. hexagona		-3.32	118.23	0.62	**0**
M. jacobus		4.3	-1.56	1	**0.01**
M. kuntee		0.00014	2.5	0	0.98
M. murdjan		-0.99	102.3	0.13	0.17
M. pralinia		0.85	63.18	0.1	0.11
M. seychellensis		2.31	13.88	0.12	0.5
M. violacea		2.1	40.07	0.53	**0**
M. vittata		2.01	63.77	0.3	0.21
N. argenteus		1.74	36.19	0.37	0.06
N. coruscum		4.27	-20.81	0.21	0.54
N. diadema		-0.01	3.49	0.6	**0.003**
N. microstoma		0.38	52.66	0	0.87
N. opercularis		1.38	55.07	0.33	0.11
N. puntatissimum		3.05	-13.42	0.67	0.09
N. sammara		0.02	3.6	0.18	**0.032**
S. caudimaculatum		0.95	45.24	0.11	0.13
S. cornutum		1.78	25.59	0.03	0.73
S. dorsomaculatum		-2.8	86.6	1	
S. melanospilos		9.08	-118.93	0.43	0.16
S. praslin		-0.43	65.04	0.13	0.42
S. rubrum		-2.8	108.19	0.3	0.26
S. seychellense		0.08	43.05	0	0.93
S. spiniferum		0.91	38.81	0.28	**0.01**
S. tiere		2.36	10.87	0.39	**0.04**
S. tiereoides		0.48	54.55	0.01	0.86
S. violaceum		0.26	55.8	0.04	0.75

Supplementary Table 7. Results of the regression analyses between dominant frequency (Fpeak) and size (TL) for each species. *P* values in bold are < 0.05.

Species	Formula	Slope estimate	Intercept estimate	Multiple R^2	***P***
F. marianus	Fpeak ~ TL	-37.54	939.84	0.74	0.14
H. adscensionis		-53.13	1800.07	0.91	0.2
H. rufus		-16.66	750.58	0.68	0.17
M. adusta		-7.68	334.51	0.65	**0**
M. amaena		-0.68	297.3	0.01	0.78
M. berndti		-10.09	420.51	0.37	**0**
M. hexagona		-0.0057	4.12	0.57	**0.004**
M. jacobus		-7.51	359.41	0.97	0.11
M. kuntee		-0.0048	3.77	0.39	**0**
M. murdjan		-8.81	397.74	0.59	**0**
M. pralinia		-7.36	364.72	0.46	**0**
M. seychellensis		-16.61	566.17	0.2	0.37
M. violacea		-10.44	359.02	0.62	**0**
M. vittata		-6.21	393.89	0.44	0.11
N. argenteus		-36.11	900.17	0.7	**0**
N. coruscum		-44.88	936.47	0.89	0.06
N. diadema		-0.042	6.48	0.47	**0.001**
N. microstoma		-17.21	628.92	0.48	**0.03**
N. opercularis		-16.56	621.07	0.89	**0**
N. puntatissimum		-43.19	864.08	0.69	0.08
N. sammara		-23.68	756.8	0.68	**0**
S. caudimaculatum		-7.26	401.27	0.3	**0.01**
S. cornutum		-26.67	677.25	0.4	0.18
S. dorsomaculatum		224.8	-2440.6	1	
S. melanospilos		-20.67	713.65	0.58	0.08
S. praslin		-6.92	431.21	0.72	**0.02**
S. rubrum		-4.06	345.45	0.07	0.61
S. seychellense		-6.73	392.55	0.27	0.23
S. spiniferum		-1.72	274.62	0.11	0.1
S. tiere		-2.72	307.4	0.15	0.25
S. tiereoides		16.02	168.09	0.04	0.66
S. violaceum		-3.15	398.54	0.4	0.26

Supplementary Table 8. Results of the regression analyses between the number of pulses in sounds and size (TL) for each species. *P* values in bold are < 0.05.

Species	Formula	Slope estimate	Intercept estimate	Multiple R^2	***P***
F. marianus		-0.01	1.42	0.01	0.89
H. adscensionis		0.37	-5.63	0.53	0.48
H. rufus		0.15	2.5	0.79	0.11
M. adusta		-0.08	7.59	0.1	0.16
M. amaena		-0.21	10.6	0.13	0.16
M. berndti		-0.03	7.56	0.01	0.71
M. hexagona		-0.22	3.15	0.018	0.68
M. jacobus		0.04	2.88	0.44	0.54
M. kuntee		-0.48	3.25	0.064	0.22
M. murdjan		-0.26	10.97	0.43	**0.01**
M. pralinia		0.04	5.52	0.04	0.32
M. seychellensis		0.03	3.68	0	0.93
M. violacea		-0.05	6.96	0.05	0.33
M. vittata		0.07	6.21	0.06	0.6
N. argenteus		-0.07	6.7	0.06	0.48
N. coruscum		0.13	0.27	0.03	0.82
N. diadema	Npulses ~ TL	0.04	1.11	0.01	0.6
N. microstoma		-0.07	5.96	0.01	0.75
N. opercularis		0.12	5.22	0.13	0.35
N. puntatissimum		0	1.08	0	0.97
N. sammara		0	5.39	0	0.96
S. caudimaculatum		0.04	3.15	0.03	0.41
S. cornutum		-0.59	12.48	0.04	0.71
S. dorsomaculatum		0	5		
S. melanospilos		0.59	-7.21	0.16	0.43
S. praslin		-0.18	8.4	0.59	**0.04**
S. rubrum		-0.11	5.62	0.16	0.43
S. seychellense		-0.03	2.4	0.05	0.62
S. spiniferum		0.07	1.48	0.38	**0**
S. tiere		0.06	0.83	0.26	0.11
S. tiereoides		0.06	3.84	0.02	0.72
S. violaceum		0.01	4.91	0.01	0.9

Supplementary Table 9. Results of the regression analyses between the duration of the last pulse in sounds (Lastpu) and size (TL) for each species. *P* values in bold are < 0.05.

Species	Formula	Slope estimate	Intercept estimate	Multiple R^2	*P*
F. marianus		-0.41	19.22	0.12	0.77
H. adscensionis		0.38	-0.22	0.44	0.54
H. rufus		0.46	0.87	0.8	0.11
M. adusta		0.26	11.4	0.11	0.15
M. amaena		1.15	4.58	0.37	**0.01**
M. berndti		0.14	16.11	0.01	0.62
M. hexagona		-1.61	45.69	0.63	**0**
M. jacobus		0.32	5.33	0.52	0.48
M. kuntee		-0.02	17.59	0	0.95
M. murdjan		0.86	6.25	0.38	**0.01**
M. pralinia		0.3	14.71	0.04	0.34
M. seychellensis		-1.08	40.35	0.13	0.49
M. violacea		1.27	-1.49	0.74	**0**
M. vittata		-0.45	24.64	0.18	0.34
N. argenteus		1.55	-4	0.77	**0**
N. coruscum	Lastpu ~ TL	-0.1	11.75	0.01	0.94
N. diadema		0.81	9.03	0.08	0.19
N. microstoma		0.87	0.69	0.66	**0**
N. opercularis		-0.16	18.65	0.02	0.73
N. sammara		0.72	3.84	0.52	**0**
S. caudimaculatum		-0.01	20.74	0	0.97
S. cornutum		10.48	-134.34	0.99	**0**
S. dorsomaculatum		-8	115.8	1	
S. melanospilos		0.38	7.97	0.04	0.72
S. praslin		0.15	15.27	0.03	0.69
S. rubrum		-1.5	52.72	0.25	0.31
S. seychellense		0.49	21.14	0.08	0.54
S. spiniferum		0.03	24.9	0	0.87
S. tiere		0.02	27.34	0	0.95
S. tiereoides		0.16	15.29	0	0.92
S. violaceum		0.08	13.16	0.23	0.42

Supplementary Table 10. Results of the Kruskal-Wallis test on the acoustical variables of sounds between all Holocentridae genera. *P* values in bold are significant. *Df* = degree of freedom. Du = sound duration, Npulses = number of pulses in sounds, F0 = fundamental frequency, Fpeak = dominant frequency, Lastpu = duration of the last pulse in sounds, Nblocks = number of blocks in sounds.

**Variables**	χ^2	*df*	***P***
Du	142.24	4	**0**
Npulses	149.93	4	**0**
F0	140.89	4	**0**
Fpeak	60.46	4	**0**
Lastpu	30.56	4	**0**
Nblocks	112.59	4	**0**
Pulse period	119.43	4	**0**
% Sounds with pattern	104.71	4	**0**
Interval	6.81	2	**0.033**

Supplementary Table 11. Results of the post-hoc Dunn tests on the acoustical variables of sounds significantly different, based on Kruskal-Wallis test, between all Holocentridae genera. Significance level = α = 0.05. Significance threshold of the Dunn test (dunn.test function with parameter 'altp' = FALSE) = α/2 = 0.025. NS = non-significant. *P* values in bold are significant. Du = sound duration, Npulses = number of pulses in sounds, F0 = fundamental frequency, Fpeak = dominant frequency, Lastpu = duration of the last pulse in sounds, Nblocks = number of blocks in sounds.

**Du**	*Flammeo*	*Holocentrus*	*Myripristis*	*Neoniphon*
Holocentrus	NS			
Myripristis	**0.000**	**0.000**		
Neoniphon	**0.018**	NS	**0.000**	
Sargocentron	**0.017**	NS	**0.000**	NS
**Npulses**	*Flammeo*	*Holocentrus*	*Myripristis*	*Neoniphon*
Holocentrus	NS			
Myripristis	**0.000**	**0.010**		
Neoniphon	**0.008**	NS	**0.000**	
Sargocentron	NS	NS	**0.000**	**0.001**
**F0**	*Flammeo*	*Holocentrus*	*Myripristis*	*Neoniphon*
Holocentrus	NS			
Myripristis	NS	NS		
Neoniphon	NS	NS	NS	
Sargocentron	NS	**0.000**	**0.000**	**0.000**

**Fpeak**	***Flammeo***	***Holocentrus***	***Myripristis***	***Neoniphon***
Holocentrus	NS			
Myripristis	NS	NS		
Neoniphon	NS	NS	**0.000**	
Sargocentron	NS	NS	**0.003**	**0.000**
**Lastpu**	*Flammeo*	*Holocentrus*	*Myripristis*	*Neoniphon*
Holocentrus	NS			
Myripristis	NS	**0.000**		
Neoniphon	NS	**0.000**	**0.019**	
Sargocentron	NS	**0.000**	NS	**0.012**
**Nblocks**	*Flammeo*	*Holocentrus*	*Myripristis*	*Neoniphon*
Holocentrus	NS			
Myripristis	**0.009**	**0.000**		
Neoniphon	NS	NS	**0.000**	
Sargocentron	NS	NS	**0.000**	NS
**Pulse period**	*Flammeo*	*Holocentrus*	*Myripristis*	*Neoniphon*
Holocentrus	NS			
Myripristis	NS	NS		
Neoniphon	NS	NS	NS	
Sargocentron	NS	**0.001**	**0.000**	**0.000**
**% Sounds with pattern**	*Flammeo*	*Holocentrus*	*Myripristis*	*Neoniphon*
Holocentrus	NS			
Myripristis	**0.006**	**0.000**		
Neoniphon	NS	NS	**0.000**	
Sargocentron	NS	NS	**0.000**	NS
**Interval**	*Myripristis*	*Neoniphon*		
Neoniphon	NS			
Sargocentron	NS	NS		

Supplementary Table 12. Results of the Kruskal-Wallis test on the acoustical variables of sounds between Holocentrinae genera. *P* values in bold are significant. *Df* = degree of freedom. Du = sound duration, Npulses = number of pulses in sounds, F0 = fundamental frequency, Fpeak = dominant frequency, Lastpu = duration of the last pulse in sounds.

**Variables**	χ^2	***df***	***P***
Du	18.04	3	**0**
Npulses	19.90	3	**0**
F0	87.61	3	**0**
Fpeak	59.03	3	**0**
Lastpu	31.59	3	**0**
Pulse period	81.57	3	**0**

Supplementary Table 13. Results of the post-hoc Dunn tests on the acoustical variables of sounds significantly different, based on Kruskal-Wallis test, between Holocentrinae genera. Significance level = α = 0.05. Significance threshold of the Dunn test (dunn.test function with parameter 'altp' = FALSE) = α/2 = 0.025. NS = non-significant. *P* values in bold are significant. Du = sound duration, Npulses = number of pulses in sounds, F0 = fundamental frequency, Fpeak = dominant frequency, Lastpu = duration of the last pulse in sounds. Values highlighted in light red are new significant differences between pairs with respect to Dunn tests carried out on all genera combined (i.e., including *Myripristis*).

**Du**	*Flammeo*	*Holocentrus*	*Neoniphon*
Holocentrus	NS		
Neoniphon	**0.002**	NS	
Sargocentron	**0.001**	**0.012**	NS
**Npulses**	*Flammeo*	*Holocentrus*	*Neoniphon*
Holocentrus	**0.003**		
Neoniphon	**0.001**	NS	
Sargocentron	**0.006**	NS	0.003
**F0**	*Flammeo*	*Holocentrus*	*Neoniphon*
Holocentrus	NS		
Neoniphon	NS	NS	
Sargocentron	NS	**0.000**	**0.000**
**Fpeak**	*Flammeo*	*Holocentrus*	*Neoniphon*
Holocentrus	NS		
Neoniphon	NS	NS	
Sargocentron	NS	NS	**0.000**
**Lastpu**	*Flammeo*	*Holocentrus*	*Neoniphon*
Holocentrus	NS		
Neoniphon	NS	**0.000**	
Sargocentron	NS	**0.000**	**0.004**
**Pulse period**	*Flammeo*	*Holocentrus*	*Neoniphon*
Holocentrus	NS		
Neoniphon	NS	NS	
Sargocentron	NS	**0.001**	**0.000**

Supplementary Table 14. Summary of the number of species (nSP), number of individuals (N) and convex hull volumes occupied by the different groups in the 3D PCAs. Relative convex hull volumes correspond to absolute convex hull volumes divided by nSP and by N.

3D PCA	Group	nSP	N	Absolute convex hull volume	Relative convex hull volume / nSP	Relative convex hull volume / N
1	Myripristinae	11	176	120.5	10.96	0.68
	Holocentrinae	20	189	47.9	2.4	0.25
2	*Myripristis*	11	176	120.5	10.96	0.68
	Sargocentron	11	103	18.5	1.68	0.18
	Holocentrus	2	7	0.2	0.1	0.03
	Neoniphon	6	77	23.1	3.85	0.30
	Flammeo	1	23	-	-	-
3	*Myripristis* species of group 1	4	61	34.4	8.6	0.56
	Myripristis species of group 2	7	115	121.8	17.4	1.06

Supplementary Table 15. Summary of the total number of sounds (n) and the mean percentages of sounds with and without pattern per species. n = number of sounds.

Species	Group	n	% Sounds without pattern (n)	% Sounds with pattern (n)
M. jacobus	1	60	100 (60)	0
M. kuntee	1	500	80 (398)	20 (102)
M. pralinia	1	520	98 (512)	2 (8)
M. vittata	1	140	99 (139)	1 (1)
M. adusta	2	420	32 (135)	68 (285)
M. amaena	2	340	52 (178)	48 (162)
M. berndti	2	420	57 (241)	43 (179)
M. hexagona	2	240	48 (114)	52 (126)
M. murdjan	2	320	36 (114)	64 (206)
M. seychellensis	2	119	17 (20)	83 (99)
M. violacea	2	440	41 (181)	59 (259)

Supplementary Table 16. Results of the post-hoc Tukey's test, following the ANOVA on the fundamental frequency of *Myripristis* species of group 1 sounds. NS = non-significant. *P* values in bold are significant.

**F0**	jac	pral	vitt
kun	NS		
pral	**0.022**	NS	
vitt	NS	NS	NS

Supplementary Table 17. Results of the Kruskal-Wallis test on the acoustical variables of sounds between *Myripristis* species of group 1. NS = non-significant. *Df* = degree of freedom. Du = sound duration, Npulses = number of pulses in sounds, Fpeak = dominant frequency, Lastpu = duration of the last pulse in sounds.

**Variables**	χ^2	***df***	***P***
Du	6.01	3	NS
Npulses	5.80	3	NS
Fpeak	1.50	3	NS
Lastpu	3.09	3	NS
Pulse period	6.32	3	NS

Supplementary Table 18. Results of the Kruskal-Wallis test on the acoustical variables of sounds between *Myripristis* species of group 2. *Df* = degree of freedom. F0 = fundamental frequency, Fpeak = dominant frequency, Lastpu = duration of the last pulse in sounds, Nblocks = number of blocks in sounds, Npulses in blocks = number of pulses in blocks. NS = non-significant. *P* values in bold are significant.

**Variables**	χ^2	***df***	***P***
F0	31.40	6	**0**
Fpeak	16.05	6	**0.013**
Lastpu	13.26	6	**0.039**
Nblocks	11.14	6	NS
Pulse period	43.37	6	**0**
% Sounds with pattern	11.9	6	NS
Interval	20.79	6	**0.002**
Npulses in blocks	21.81	6	**0.001**

Supplementary Table 19. Results of the ANOVA tests on sound duration and the number of pulses in sounds between *Myripristis* species of group 2. *Df* = degree of freedom. Du = sound duration, Npulses = number of pulses in sounds. *P* values in bold are significant.

**Variables**	**F**	***df***	***P***
Du	5.51	6	0.000
Npulses	3.29	6	0.005

Supplementary Table 20. Results of the post-hoc Dunn tests on the acoustical variables of sounds significantly different, based on Kruskal-Wallis tests, between *Myripristis* species of group 2. Significance level = α = 0.05. Significance threshold of the Dunn test (dunn.test function with parameter 'altp' = FALSE) = α/2 = 0.025. NS = non-significant. *P* values in bold are significant. F0 = fundamental frequency, Fpeak = dominant frequency, Lastpu = duration of the last pulse in sounds, Nblocks = number of blocks in sounds, Npulses in blocks = number of pulses in blocks.

**F0**	adu	ama	bern	hex	mur	sey
ama	**0.002**					
bern	**0.001**	NS				
hex	NS	**0.010**	**0.010**			
mur	**0.000**	NS	NS	**0.004**		
sey	NS	**0.015**	**0.016**	NS	**0.009**	
viol	**0.010**	NS	NS	NS	NS	NS
**Fpeak**	adu	ama	bern	hex	mur	sey
ama	NS					
bern	NS	NS				
hex	NS	**0.026**	NS			
mur	NS	NS	NS	NS		
sey	**0.022**	**0.005**	**0.012**	NS	**0.024**	
viol	NS	NS	NS	NS	NS	**0.028**
**Lastpu**	adu	ama	bern	hex	mur	sey
ama	NS					
bern	NS	NS				
hex	NS	NS	NS			
mur	NS	NS	NS	NS		
sey	NS	**0.023**	NS	NS	NS	
viol	NS	NS	NS	NS	NS	NS

**Pulse period**	adu	ama	bern	hex	mur	sey
ama	**0.000**					
bern	**0.000**	NS				
hex	NS	**0.000**	**0.000**			
mur	**0.000**	NS	NS	**0.000**		
sey	NS	NS	NS	NS	NS	
viol	**0.002**	NS	NS	**0.005**	NS	NS
**Interval**	adu	ama	bern	hex	mur	sey
ama	NS					
bern	NS	NS				
hex	NS	NS	NS			
mur	NS	NS	NS	NS		
sey	**0.001**	**0.001**	**0.018**	**0.001**	**0.019**	
viol	NS	NS	NS	NS	NS	**0.001**
**Npulses in blocks**	adu	ama	bern	hex	mur	sey
ama	NS					
bern	NS	NS				
hex	NS	NS	NS			
mur	NS	NS	NS	NS		
sey	**0.013**	**0.001**	**0.001**	**0.016**	**0.001**	
viol	NS	NS	NS	NS	NS	**0.013**

Supplementary Table 21. Results of the post-hoc Tukey's test on the sound duration and the number of pulses in sounds, significantly different, based on ANOVA tests, between *Myripristis* species of group 2. Du = sound duration, Npulses = number of pulses in sounds. NS = non-significant. *P* values in bold are significant.

**Du**	adu	ama	bern	hex	mur	sey
ama	**0.027**					
bern	NS	NS				
hex	NS	**0.002**	NS			
mur	NS	NS	NS	NS		
sey	NS	**0.000**	**0.025**	NS	**0.010**	
viol	NS	**0.034**	NS	NS	NS	NS
**Npulses**	adu	ama	bern	hex	mur	sey
ama	NS					
bern	NS	NS				
hex	NS	NS	NS			
mur	NS	NS	NS	NS		
sey	NS	**0.003**	**0.048**	NS	**0.043**	
viol	NS	NS	NS	NS	NS	NS

Supplementary Table 22. Results of the Kruskal-Wallis test on the acoustical variables of sounds between *Sargocentron* species. *P* values in bold are significant. *Df* = degree of freedom. Du = sound duration, Npulses = number of pulses in sounds, F0 = fundamental frequency, Fpeak = dominant frequency, Lastpu = duration of the last pulse in sounds.

**Variables**	χ^2	***df***	***P***
Du	27.11	10	0.003
Npulses	60.19	10	0
F0	85.31	10	0
Fpeak	28.21	10	0.002
Lastpu	37.55	10	0
Pulse period	81.19	10	0

Supplementary Table 23. Results of the post-hoc Dunn tests on the acoustical variables of sounds significantly different, based on Kruskal-Wallis test, between *Sargocentron* species. Significance level = α = 0.05. Significance threshold of the Dunn test (dunn.test function with parameter 'altp' = FALSE) = α/2 = 0.025. NS = non-significant. *P* values in bold are significant. Du = sound duration, Npulses = number of pulses in sounds, F0 = fundamental frequency, Fpeak = dominant frequency, Lastpu = duration of the last pulse in sounds.

**Du**	cau	corn	dorso	mel	pra	rub	seyc	spi	tiere	tiereoides
corn	NS									
dorso	NS	NS								
mel	NS	NS	NS							
pra	NS	NS	NS	NS						
rub	NS	NS	NS	NS	NS					
seyc	NS	NS	NS	NS	NS	NS				
spi	NS	NS	NS	NS	NS	NS	NS			
tiere	NS	NS	NS	NS	NS	NS	NS	NS		
tiereoides	NS	NS	NS	**0.010**	**0.009**	**NS**	**0.005**	**0.016**	**0.008**	
vio	NS	NS	NS	NS	NS	NS	NS	NS	NS	NS

**Npulses**	cau	corn	dorso	mel	pra	rub	seyc	spi	tiere	tiereoides
corn	NS									
dorso	NS	NS								
mel	NS	NS	NS							
pra	NS	NS	NS	NS						
rub	NS	NS	NS	NS	NS					
seyc	**0.004**	**0.017**	**0.003**	NS	**0.010**	**0.025**				
spi	**0.021**	NS	**0.013**	NS	NS	NS	NS			
tiere	**0.000**	**0.003**	**0.001**	**0.013**	**0.001**	**0.005**	NS	0.021		
tiereoides	**0.019**	NS	NS	NS	NS	NS	**0.000**	**0.000**	**0.000**	
vio	NS	NS	NS	NS	NS	NS	**0.001**	**0.004**	**0.000**	NS

**F0**	cau	corn	dorso	mel	pra	rub	seyc	spi	tiere	tiereoides
corn	**0.002**									
dorso	**0.017**	NS								
mel	NS	NS	NS							
pra	**0.006**	NS	NS	NS						
rub	**0.016**	NS	NS	NS	NS					
seyc	NS	**0.005**	**0.021**	NS	**0.016**	NS				
spi	NS	**0.000**	**0.003**	**0.022**	**0.000**	**0.001**	NS			
tiere	**0.001**	**0.000**	**0.000**	**0.001**	**0.000**	**0.000**	**0.011**	**0.021**		
tiereoides	**0.004**	NS	NS	NS	NS	NS	**0.016**	**0.000**	**0.000**	
vio	**0.004**	NS	NS	NS	NS	NS	**0.016**	**0.000**	**0.000**	NS

**Fpeak**	cau	corn	dorso	mel	pra	rub	seyc	spi	tiere	tiereoides
corn	NS									
dorso	NS	NS								
mel	NS	NS	NS							
pra	NS	NS	NS	NS						
rub	NS	NS	NS	NS	NS					
seyc	NS	NS	NS	NS	NS	NS				
spi	NS	NS	NS	NS	NS	NS	NS			
tiere	NS	NS	NS	NS	NS	NS	NS	NS		
tiereoides	**0.015**	NS	NS	NS	**0.017**	NS	NS	**0.002**	**0.006**	
vio	NS	NS	NS	NS	NS	NS	NS	NS	NS	NS

**Lastpu**	cau	corn	dorso	mel	pra	rub	seyc	spi	tiere	tiereoides
corn	NS									
dorso	NS	NS								
mel	NS	NS	NS							
pra	NS	NS	NS	NS						
rub	NS	NS	NS	**0.008**	**0.017**					
seyc	**0.010**	NS	NS	**0.001**	**0.002**	NS				
spi	NS	NS	NS	**0.002**	**0.007**	NS	NS			
tiere	NS	NS	NS	**0.002**	**0.007**	NS	NS	NS		
tiereoides	NS	NS	NS	NS	NS	NS	NS	NS	NS	
vio	NS	NS	NS	NS	NS	NS	**0.006**	NS	NS	NS

**Pulse period**	cau	corn	dorso	mel	pra	rub	seyc	spi	tiere	tiereoides
corn	**0.002**									
dorso	**0.024**	NS								
mel	NS	NS	NS							
pra	**0.002**	NS	NS	NS						
rub	NS	NS	NS	NS	NS					
seyc	NS	**0.003**	**0.022**	NS	**0.004**	NS				
spi	NS	**0.000**	**0.006**	NS	**0.000**	**0.003**	NS			
tiere	**0.000**	**0.000**	**0.000**	**0.000**	**0.000**	**0.000**	**0.017**	**0.015**		
tiereoides	**0.013**	NS	NS	NS	NS	NS	**0.018**	**0.001**	**0.000**	
vio	0.012	NS	NS	NS	NS	NS	**0.017**	**0.001**	**0.000**	NS

Supplementary Table 24. Results of the Kruskal-Wallis test on the acoustical variables of sounds between *Neoniphon* species. *P* values in bold are significant. *Df* = degree of freedom. Du = sound duration, F0 = fundamental frequency, Fpeak = dominant frequency, Lastpu = duration of the last pulse in sounds.

**Variables**	χ^2	***df***	***P***
Du	51.69	6	0
F0	47.95	5	0
Fpeak	15.78	6	0.015
Lastpu	22.07	6	0.001
Pulse period	46.96	6	0

Supplementary Table 25. Results of the post-hoc Dunn tests on the acoustical variables of sounds significantly different, based on Kruskal-Wallis test, between *Neoniphon* species. Significance level = α = 0.05. Significance threshold of the Dunn test (dunn.test function with parameter 'altp' = FALSE) = α/2 = 0.025. NS = non-significant. *P* values in bold are significant. Du = sound duration, F0 = fundamental frequency, Fpeak = dominant frequency, Lastpu = duration of the last pulse in sounds.

**Du**	arg	cor	dia	mic	oper	pun
cor	**0.001**					
dia	**0.000**	NS				
mic	NS	**0.008**	**0.001**			
oper	NS	**0.001**	**0.000**	NS		
pun	**0.000**	NS	NS	**0.003**	**0.000**	
sam	**0.009**	**0.025**	**0.002**	NS	**0.009**	**0.010**
**F0**	arg	cor	dia	mic	oper	
cor	**0.003**					
dia	**0.003**	NS				
mic	**0.003**	NS	NS			
oper	NS	NS	NS	NS		
sam	NS	**0.000**	**0.000**	**0.000**	**0.000**	
**Fpeak**	arg	cor	dia	mic	oper	pun
cor	NS					
dia	NS	NS				
mic	NS	NS	NS			
oper	NS	NS	NS	NS		
pun	NS	NS	NS	NS	NS	
sam	NS	NS	NS	NS	NS	NS

**Lastpu**	arg	cor	dia	mic	oper	pun
cor	NS					
dia	NS	NS				
mic	NS	NS	**0.002**			
oper	NS	NS	NS	NS		
pun	NS	NS	NS	NS	NS	
sam	NS	NS	**0.000**	NS	NS	NS
**Pulse period**	arg	cor	dia	mic	oper	pun
cor	**0.007**					
dia	NS	NS				
mic	**0.011**	NS	NS			
oper	NS	NS	NS	NS		
pun	NS	NS	NS	NS	NS	
sam	NS	**0.000**	**0.000**	**0.000**	**0.001**	**0.014**

Supplementary Table 26. Results of the post-hoc Tukey's test on the number of pulses in sounds, significantly different, based on an ANOVA test, between *Neoniphon* species. NS = non-significant. *P* values in bold are significant. Npulses = number of pulses in sounds.

**Npulses**	arg	cor	dia	mic	oper	pun
cor	**0.000**					
dia	**0.000**	NS				
mic	NS	**0.010**	**0.000**			
oper	NS	**0.000**	**0.000**	NS		
pun	**0.000**	NS	NS	**0.000**	**0.000**	
sam	NS	**0.003**	**0.000**	NS	NS	**0.000**

Supplementary Table 27. Summary of the number of sounds (n), individuals (N) and species (nSP) collected for each genus at each geographical area. GD = Guadeloupe, GU = Guam, H = Holocentrinae, M = Myripristinae, PF = French Polynesia, PH = Philippines, Sey = Seychelles, SF = subfamily. Asterisks (*) indicate species that had never been recorded previous to this study. The taxonomy used in this study refers to the terminology revision proposed by Dornburg *et al.* (2012).

SF	Genus (nSP)	Species	GD	PF	GU	SEY	PH	TOTAL
					n (N)			n / species (N / species)
M	*Myripristis* (11)	*M. pralinia*		260 (13)	60 (3)	80 (4)	120 (6)	**520 (26)**
		*M. vittata**		80 (4)			60 (3)	**140 (7)**
		M. kuntee		140 (7)	140 (7)	100 (5)	120 (6)	**500 (25)**
		*M. jacobus**	60 (3)					**60 (3)**
		M. berndti		140 (7)	120 (6)	20 (1)	140 (7)	**420 (21)**
		M. amaena		200 (10)	120 (6)		20 (1)	**340 (17)**
		*M. hexagona**				80 (4)	160 (8)	**240 (12)**
		*M. adusta**		160 (8)	140 (7)	120 (6)		**420 (21)**
		*M. seychellensis**				119 (6)		**119 (6)**
		*M. murdjan**			120 (6)	140 (7)	60 (3)	**320 (16)**
		M. violacea		180 (9)		140 (7)	120 (6)	**440 (22)**
H	*Sargocentron* (11)	*S. tiere*		140 (7)	80 (4)			**220 (11)**
		*S. tiereoides**		40 (2)			120 (6)	**160 (8)**
		S. caudimaculatum		119 (6)	92 (5)	120 (6)	120 (6)	**451 (23)**
		*S. violaceum**		100 (5)		20 (1)	20 (1)	**140 (7)**
		S. spiniferum		136 (7)	129 (7)	120 (6)	100 (5)	**485 (25)**
		*S. seychellense**				140 (7)		**140 (7)**
		*S. dorsomaculatum**					40 (2)	**40 (2)**
		*S. cornutum**					120 (6)	**120 (6)**
		*S. rubrum**					120 (6)	**120 (6)**
		*S. praslin**				80 (4)	60 (3)	**140 (7)**
		*S. melanospilos**				120 (6)		**120 (6)**
H	*Flammeo* (1)	*F. marianus**	76 (4)					**76 (4)**
H	*Holocentrus* (2)	*H. rufus*	80 (4)					**80 (4)**
		H. adscensionis	100 (5)					**100 (5)**
H	*Neoniphon* (8)	*N. vexillarium**	40 (2)					**40 (2)**
		N. sammara		120 (6)	120 (6)	140 (7)	120 (6)	**500 (25)**
		*N. argenteus**		120 (6)	80 (4)			**200 (10)**
		*N. coruscum**	80 (4)					**80 (4)**
		*N. punctatissimum**		91 (5)				**91 (5)**
		N. diadema		125 (7)	115 (6)	120 (6)	120 (6)	**480 (25)**
		*N. opercularis**		20 (1)	40 (2)		120 (6)	**180 (9)**
		*N. microstoma**		110 (6)	70 (5)			**180 (11)**
**TOTAL**	**n / ori (N / ori)**		**436 (22)**	**2281 (116)**	**1426 (74)**	**1659 (83)**	**1860 (93)**	**7662 sounds 388 specimens**
	**nSP / ori**		**6**	**18**	**14**	**16**	**19**	**33 species**

Supplementary Table 28. Summary of sizes (standard and total lengths, SL and TL, respectively; min and max) for each species at each geographical area. A hyphen indicates that the measurements were not taken.

Species	GD		PF		GU		SEY		PH	
	**SL** Mean ± sd [min – max]	**TL** Mean ± sd [min – max]	**SL** Mean ± sd [min – max]	**TL** Mean ± sd [min – max]	**SL** Mean ± sd [min – max]	**TL** Mean ± sd [min – max]	**SL** Mean ± sd [min – max]	**TL** Mean ± sd [min – max]	**SL** Mean ± sd [min – max]	**TL** Mean ± sd [min – max]
M. pralinia			12 ± 3.4 [6 - 14.5]	14 ± 4.4 [7.5 - 18.5]	8 ± 2.8 [5.4 - 11]	10 ± 3.7 [7 - 14.3]	13 ± 1.3 [11.5 - 14.7]	16 ± 1.7 [14.4 - 18.4]	9.1 ± 0.8 [8 - 9.9]	11 ± 1 [9.9 - 12.1]
M. vittata			13 ± 1.5 [12 - 14.8]	17 ± 1.6 [15.6 - 18.9]					7.8 ± 0.7 [7 - 8.3]	9.8 ± 0.8 [8.9 - 10.5]
M. kuntee			10 ± 2.4 [6.8 - 12.5]	13 ± 3.2 [8.3 - 16]	6.8 ± 0.8 [5.6 - 7.7]	8.5 ± 1 [6.8 - 9.8]	16 ± 0.3 [15.2 - 16]	20 ± 0.4 [19.4 - 20.5]	10 ± 1.7 [7.6 - 12.5]	13 ± 2.1 [9.5 - 15.7]
M. jacobus	-	11 ± 5 [7.7 - 17]								
M. berndti			9.5 ± 3.4 [5 - 14]	12 ± 4.4 [6.5 - 18]	8.9 ± 2.1 [5.5 - 10.8]	11 ± 2.5 [7 - 13.7]	21 [21.2 - 21.2]	26 [26.5 - 26.5]	12 ± 3.5 [7.7 - 15.9]	14 ± 4.3 [9.4 - 19.5]
M. amaena			11 ± 2.5 [5 - 14]	14 ± 3.1 [6.5 - 18]	7.1 ± 1.3 [5 - 8.5]	9 ± 1.6 [6.3 - 10.6]			12 [12.3 - 12.3]	16 [15.8 - 15.8]
M. hexagona							16 ± 0.7 [15.5 - 17]	21 ± 0.9 [19.3 - 21.2]	11 ± 1.2 [9 - 12.2]	14 ± 1.4 [11.6 - 15.4]
M. adusta			10 ± 2.1 [7.3 - 13.1]	13 ± 2.5 [9.3 - 16.4]	7.3 ± 3.5 [4.7 - 15]	9.3 ± 4.3 [6 - 18.7]	15 ± 3.8 [7.4 - 17.5]	19 ± 4.8 [9.5 - 22.2]		
M. seychellensis							17 ± 1.5 [15 - 19.2]	22 ± 1.8 [19.1 - 24]		
M. murdjan					7.1 ± 1.8 [5.2 - 10]	9.1 ± 2.4 [6.8 - 12.8]	14 ± 2.7 [9.6 - 17]	18 ± 3 [12.8 - 21.2]	12 ± 2.6 [9 - 14]	15 ± 3.2 [11.4 - 17.3]
M. violacea			6.2 ± 1.3 [4 - 8.3]	7.9 ± 1.8 [5 - 10.6]			14 ± 0.9 [13 - 15.3]	18 ± 1 [16.6 - 19.1]	11 ± 3.4 [6.8 - 16.5]	14 ± 4.2 [9.4 - 20.9]
S. tiere			14 ± 2.1 [10.5 - 16]	16 ± 2.4 [12.6 - 19]	20 ± 2.4 [17.5 - 22.7]	24 ± 2.7 [21.4 - 27.3]				
S. tiereoides			11 ± 2.5 [9.5 - 13]	14 ± 3 [11.5 - 15.8]					11 ± 0.8 [10.5 - 12.4]	13 ± 0.9 [12.6 - 14.7]
S. caudimaculatum			13 ± 0.6 [12 - 13.5]	16 ± 0.9 [14.5 - 17]	15 ± 1.3 [13.5 - 16.5]	18 ± 1.5 [16.2 - 19.7]	19 ± 1.7 [17 - 21]	23 ± 2.3 [20.4 - 25.6]	13 ± 2.9 [8 - 15]	15 ± 3.4 [9.3 - 17.7]
S. violaceum			11 ± 3.5 [8.1 - 15]	13 ± 4.4 [9.5 - 18.2]			20 [20.5 - 20.5]	24 [24.3 - 24.3]	14 [13.5 - 13.5]	16 [15.7 - 15.7]
S. spiniferum			15 ± 4.8 [8.4 - 23.7]	18 ± 5.5 [10.2 - 27.8]	9.9 ± 3.8 [4.6 - 16.5]	12 ± 4.5 [5.5 - 19.5]	19 ± 3.2 [15.7 - 24.3]	22 ± 3.6 [18.8 - 28.3]	18 ± 2.4 [15.4 - 21]	21 ± 2.8 [17.8 - 24.5]
S. seychellense							14 ± 2.4 [8.7 - 15.5]	16 ± 2.9 [10.8 - 19]		

S. dorsomaculatum									10 ± 0.7 [10 - 11]	13 ± 0.4 [12.5 - 13]
S. cornutum									13 ± 0.6 [12 - 13.3]	15 ± 0.7 [13.8 - 15.8]
S. rubrum									14 ± 1.5 [13 - 16.5]	18 ± 1.7 [16.1 - 20]
S. praslin							19 ± 0.4 [18.5 - 19.4]	23 ± 0.5 [22.2 - 23.5]	14 ± 2.3 [12.2 - 16.6]	17 ± 2.9 [14.7 - 20.1]
S. melanospilos							15 ± 0.5 [14.5 - 16]	19 ± 0.8 [17.5 - 19.7]		
F. marianus	-	16 ± 1.1 [14 - 16.5]								
H. rufus	-	12 ± 2.8 [10 - 16]								
H. adscensionis	22 ± 1 [21 - 23]	27 ± 1.7 [26 - 29]								
N. vexillarium	-	-								
N. sammara			16 ± 1.3 [14 - 17]	19 ± 1.2 [17 - 20]	8.6 ± 1.8 [5.6 - 11.2]	11 ± 2.2 [7.1 - 14]	17 ± 1.7 [14 - 19.5]	21 ± 1.9 [17 - 23.2]	11 ± 2 [8.5 - 13.5]	12 ± 2.5 [10.1 - 16]
N. argenteus			11 ± 3.4 [6.3 - 14]	13 ± 4.1 [7.3 - 16.7]	7.1 ± 2.3 [5.5 - 10.5]	8.8 ± 3 [6.8 - 13.2]				
N. coruscum	-	9.9 ± 1.1 [9 - 11.3]								
N. punctatissimum			9.5 ± 1.6 [7 - 11.5]	12 ± 2 [8.5 - 14.2]						
N. diadema			10 ± 1.5 [8.9 - 13]	12 ± 1.9 [10 - 15.5]	8.3 ± 2.6 [5.1 - 11.7]	9.9 ± 3.2 [6.1 - 14]	11 ± 0.3 [11 - 11.8]	14 ± 0.3 [13.4 - 14.2]	9.6 ± 1 [8.2 - 11.2]	12 ± 1.2 [9.9 - 13.2]
N. opercularis			18 [18.3 - 18.3]	21 [21.1 - 21.1]	14 ± 3.5 [11 - 16]	16 ± 4.2 [13 - 18.9]				
N. microstoma			14 ± 0.8 [12.5 - 14.5]	16 ± 1.2 [14.5 - 17.5]	9 ± 1.5 [7.7 - 11.2]	11 ± 1.7 [9.5 - 13.3]				

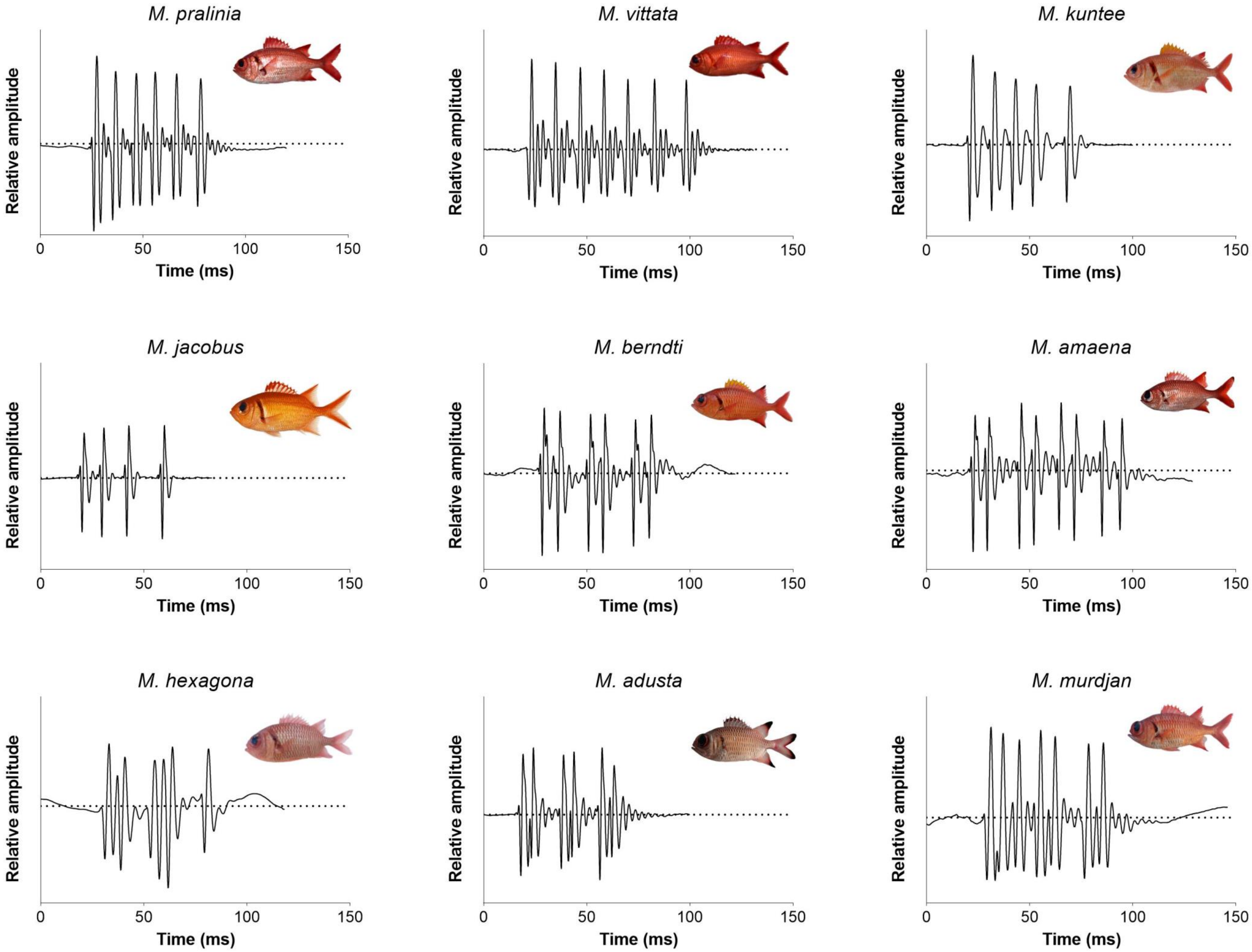

M. pralinia
M. vittata
M. kuntee
M. jacobus
M. berndti
M. amaena
M. hexagona
M. adusta
M. murdjan
Relative amplitude
Time (ms)
0
50
100
150

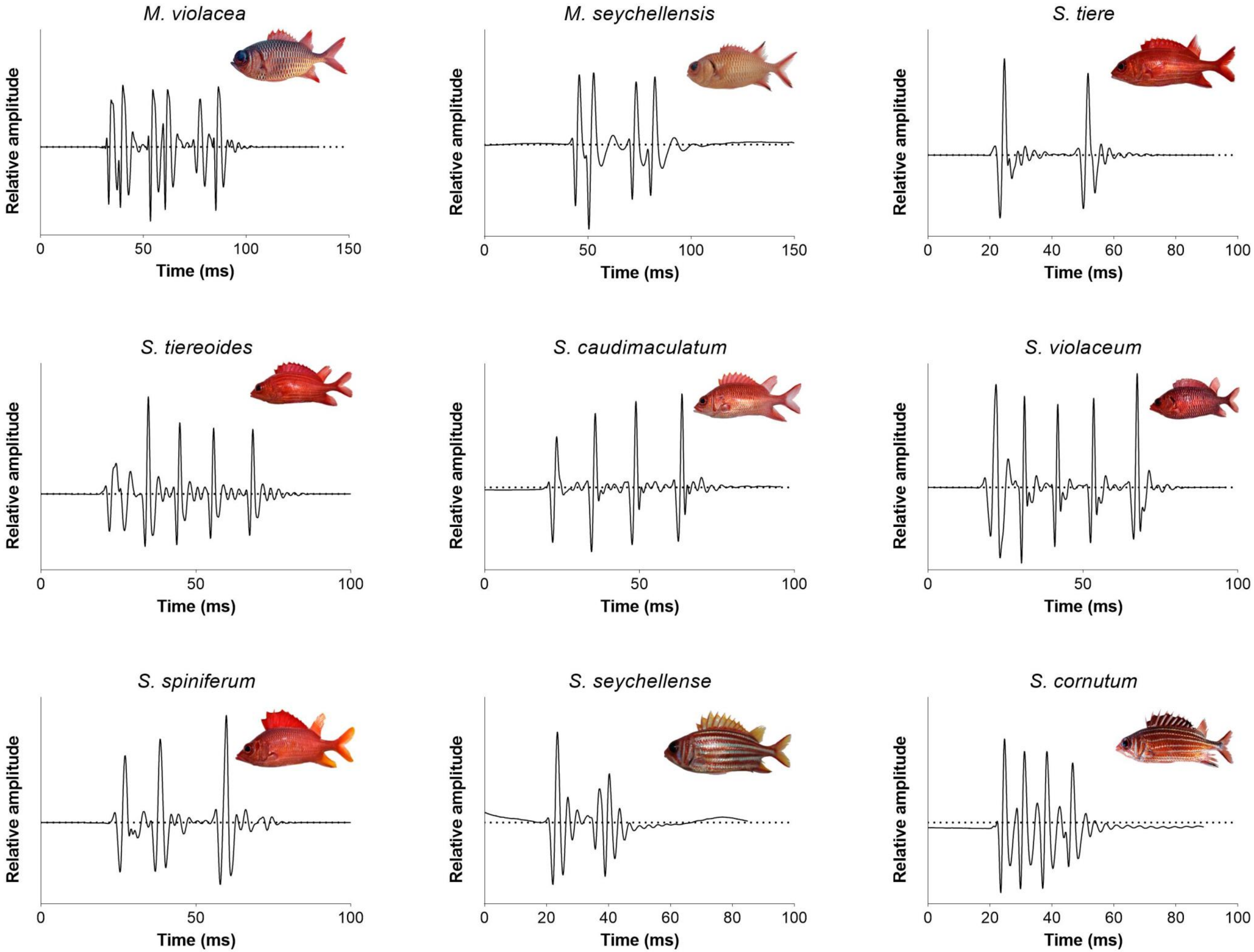

M. violacea
M. seychellensis
S. tiere
S. tiereoides
S. caudimaculatum
S. violaceum
S. spiniferum
S. seychellense
S. cornutum
Relative amplitude
Time (ms)
0
20
40
50
60
80
100
150

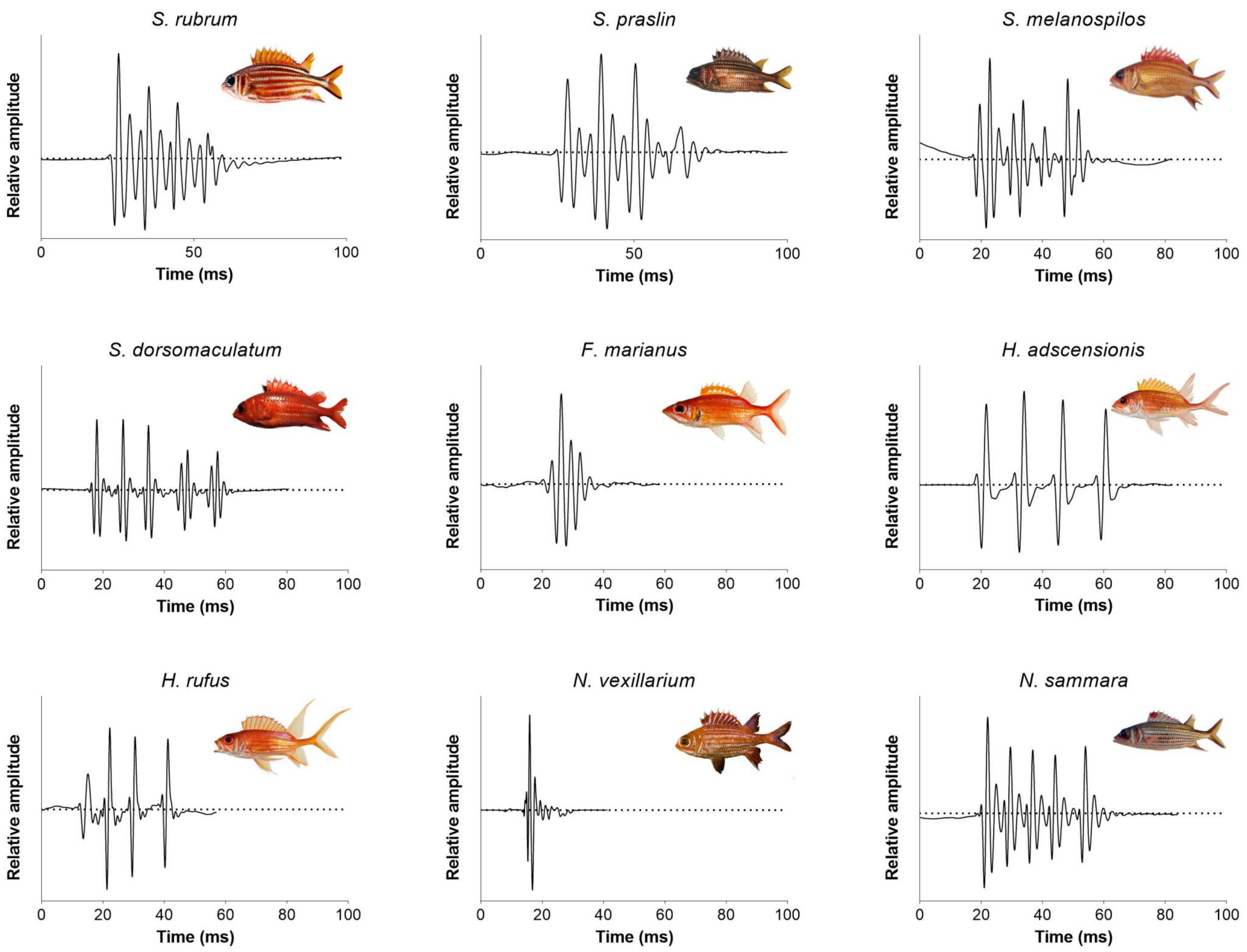
S. rubrum
S. praslin
S. melanospilos
S. dorsomaculatum
F. marianus
H. adscensionis
H. rufus
N. vexillarium
N. sammara
Relative amplitude
Time (ms)
0
20
40
50
60
80
100

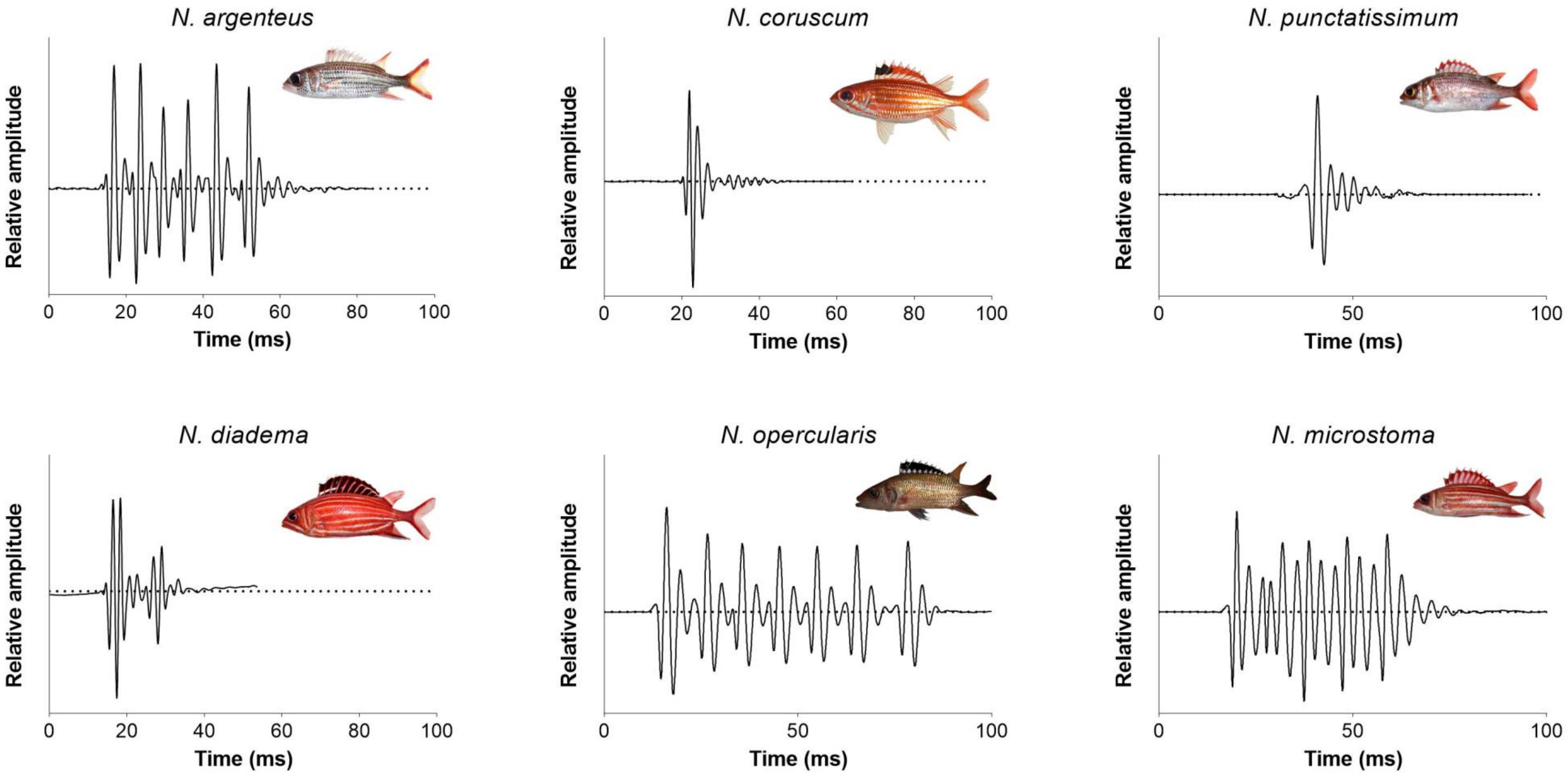

Supplementary Fig. 1.│ Oscillograms of a representative sound of each of the 33 species of Holocentridae investigated in this study.

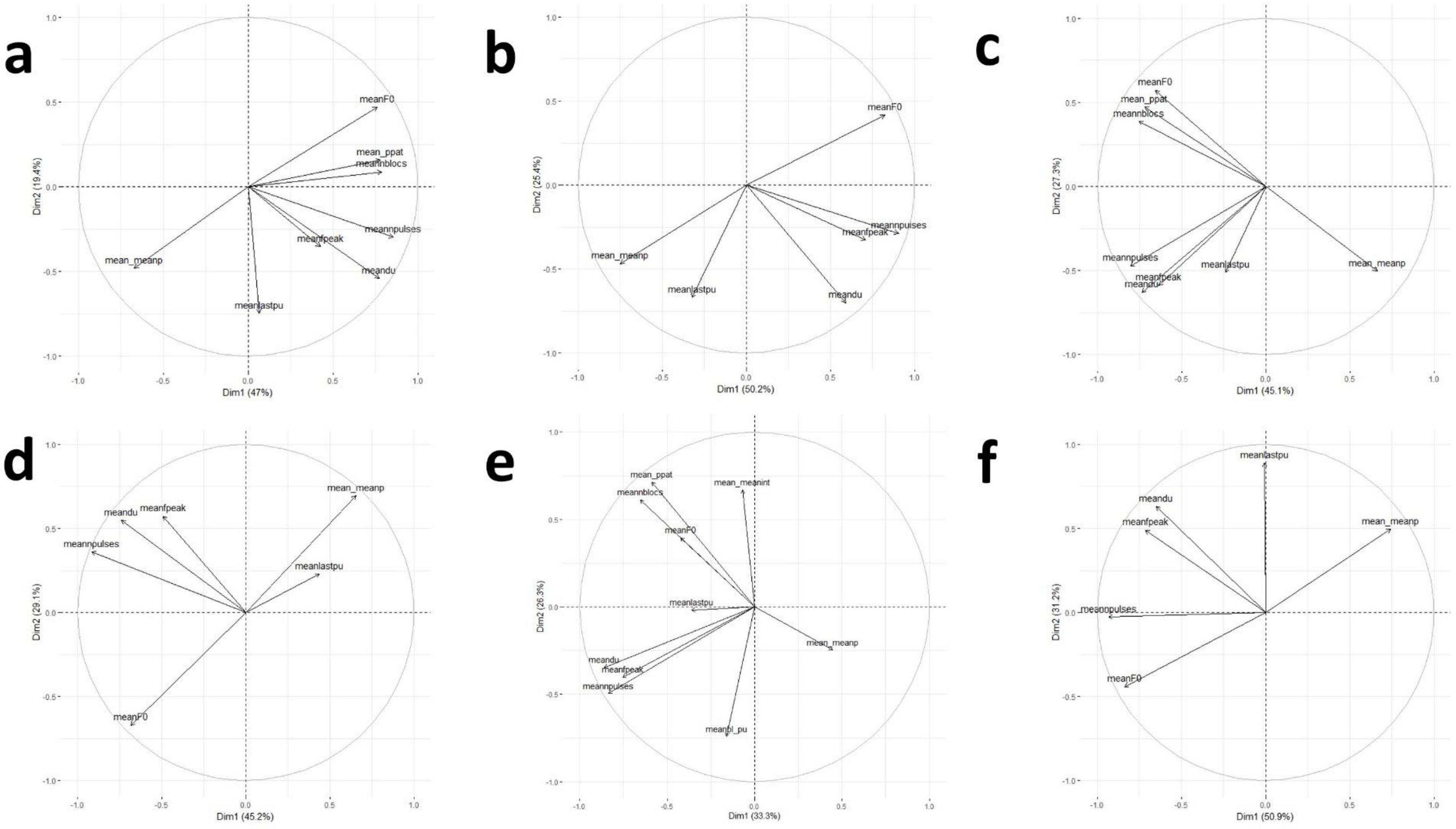

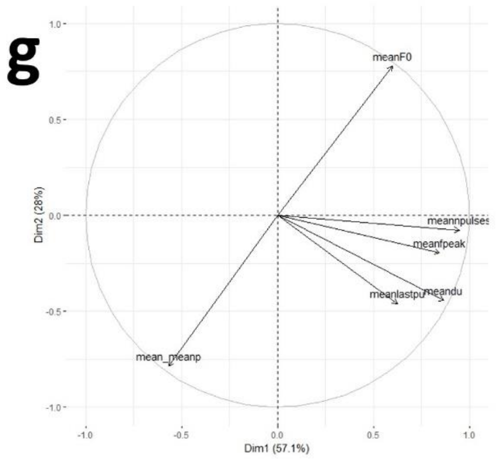

Supplementary Fig. 2.│ Variable correlation plots for principal component analyses. At the levels of a subfamily and genus, b Holocentrinae genera, c *Myripristis* species from groups 1 and 2, d *Myripristis* species from group 1, e *Myripristis* species from group 2, f *Sargocentron* species and g *Neoniphon* species.

Supplementary Video 1.│ Scatterplot of the first three principal components (PC1, PC2, PC3) performed with 6 acoustical variables (sound duration, number of pulses, fundamental and dominant frequencies, pulse period and duration of the last pulse) of the sounds produced by fishes of the family Holocentridae, based on their subfamily (N = 365). CH = convex hull.

Supplementary Video 2.│ Scatterplot of the first three principal components (PC1, PC2, PC3) performed with 6 acoustical variables (sound duration, number of pulses, fundamental and dominant frequencies, pulse period and duration of the last pulse) of the sounds produced by fishes of the family Holocentridae, based on their genus (N = 365). CH = convex hull.

Supplementary Video 3.│ Scatterplot of the first three principal components (PC1, PC2, PC3) performed with 8 acoustical variables (sound duration, number of pulses, fundamental and dominant frequencies, pulse period and duration of the last pulse, number of blocks in sounds, percentage of sounds with pattern) of the sounds produced by *Myripristis* species, based on their group (N = 176). CH = convex hull.

Supplementary Video 4.│ Scatterplot of the first three principal components (PC1, PC2, PC3) performed with the 10 acoustical variables (sound duration, number of pulses, fundamental and dominant frequencies, pulse period and duration of the last pulse, number of blocks in sounds, number of pulses in blocks, interval, percentage of sounds with pattern) of the sounds produced by *Myripristis* species of group 2, based on the species (N = 101). CH = convex hull.
